# A highly visible-transparent thermochromic smart window with broadband infrared modulation for all-season energy savings

**DOI:** 10.1093/nsr/nwae408

**Published:** 2024-11-13

**Authors:** Yi Jiang, Yunlong Wang, Deshuo Kong, Zipeng Chen, Zhengwei Yang, Ningning Cao, Haowen Chi, Shining Zhu, Qiuhong Zhang, Jia Zhu, Bin Zhu

**Affiliations:** National Laboratory of Solid State Microstructures, College of Engineering and Applied Sciences, Jiangsu Key Laboratory of Artificial Functional Materials, Frontiers Science Center for Critical Earth Material Cycling, Collaborative Innovation Center of Advanced Microstructures, Nanjing University, Nanjing 210093, China; National Laboratory of Solid State Microstructures, College of Engineering and Applied Sciences, Jiangsu Key Laboratory of Artificial Functional Materials, Frontiers Science Center for Critical Earth Material Cycling, Collaborative Innovation Center of Advanced Microstructures, Nanjing University, Nanjing 210093, China; Key Laboratory of High Performance Polymer Material and Technology of MOE, Department of Polymer Science and Engineering, School of Chemistry and Chemical Engineering, Nanjing University, Nanjing 210093, China; National Laboratory of Solid State Microstructures, College of Engineering and Applied Sciences, Jiangsu Key Laboratory of Artificial Functional Materials, Frontiers Science Center for Critical Earth Material Cycling, Collaborative Innovation Center of Advanced Microstructures, Nanjing University, Nanjing 210093, China; National Laboratory of Solid State Microstructures, College of Engineering and Applied Sciences, Jiangsu Key Laboratory of Artificial Functional Materials, Frontiers Science Center for Critical Earth Material Cycling, Collaborative Innovation Center of Advanced Microstructures, Nanjing University, Nanjing 210093, China; National Laboratory of Solid State Microstructures, College of Engineering and Applied Sciences, Jiangsu Key Laboratory of Artificial Functional Materials, Frontiers Science Center for Critical Earth Material Cycling, Collaborative Innovation Center of Advanced Microstructures, Nanjing University, Nanjing 210093, China; National Laboratory of Solid State Microstructures, College of Engineering and Applied Sciences, Jiangsu Key Laboratory of Artificial Functional Materials, Frontiers Science Center for Critical Earth Material Cycling, Collaborative Innovation Center of Advanced Microstructures, Nanjing University, Nanjing 210093, China; National Laboratory of Solid State Microstructures, College of Engineering and Applied Sciences, Jiangsu Key Laboratory of Artificial Functional Materials, Frontiers Science Center for Critical Earth Material Cycling, Collaborative Innovation Center of Advanced Microstructures, Nanjing University, Nanjing 210093, China; Key Laboratory of High Performance Polymer Material and Technology of MOE, Department of Polymer Science and Engineering, School of Chemistry and Chemical Engineering, Nanjing University, Nanjing 210093, China; National Laboratory of Solid State Microstructures, College of Engineering and Applied Sciences, Jiangsu Key Laboratory of Artificial Functional Materials, Frontiers Science Center for Critical Earth Material Cycling, Collaborative Innovation Center of Advanced Microstructures, Nanjing University, Nanjing 210093, China; National Laboratory of Solid State Microstructures, College of Engineering and Applied Sciences, Jiangsu Key Laboratory of Artificial Functional Materials, Frontiers Science Center for Critical Earth Material Cycling, Collaborative Innovation Center of Advanced Microstructures, Nanjing University, Nanjing 210093, China

**Keywords:** thermochromic smart window, visible transparency, infrared modulation, two-way shape memory polymer

## Abstract

Thermochromic smart windows effectively reduce the energy consumption for buildings through passive light modulation including the transmission of visible (T_Vis_) and near-infrared (T_NIR_) light, and the emissivity of mid-infrared (ε_MIR_) light in response to ambient temperature change. However, thermochromic windows that maintain high T_Vis_ while modulating T_NIR_ and ε_MIR_ simultaneously are highly desirable but still challenging. Here, we develop a thermochromic smart window based on a two-way shape memory polymer to enable reversible transformation and achieve T_NIR_ modulation of 44.0% and ε_MIR_ modulation of 76.5% while maintaining high T_Vis_ (>50%). Compared to traditional windows based on silica glass, this device shows 4°C lower temperature in summer daytime, 2°C higher in winter daytime, and 1°C higher in spring nighttime. It is expected that our device can achieve greater annual energy savings in comparison with commercial glass anywhere in the world and promote the progress of thermochromic windows for energy-efficient buildings.

## Teaser

A highly visible-transparent thermochromic smart window based on a two-way shape memory polymer is developed with T_NIR_ modulation of 44.0% and ε_MIR_ modulation of 76.5% for all season energy efficient buildings.

## INTRODUCTION

Developing energy-efficient buildings is essential for global carbon neutrality, as 40% of global energy consumption is used in buildings [1–3]. Windows, essential components for occupants’ comfort, are considered among the least energy-efficient parts [4], calling for significant advancement in the field of smart windows with dynamic thermal radiation tuning, adaptive to the variable environment. Previously, significant efforts have been made on smart windows based on electro-, thermo-, mechano-, and photo-responses [5,6]. Among them, thermochromic smart windows are considered an attractive energy-efficient passive auto-switching device due to their zero-energy-consumption characteristics [7,8].

**Figure 1.**
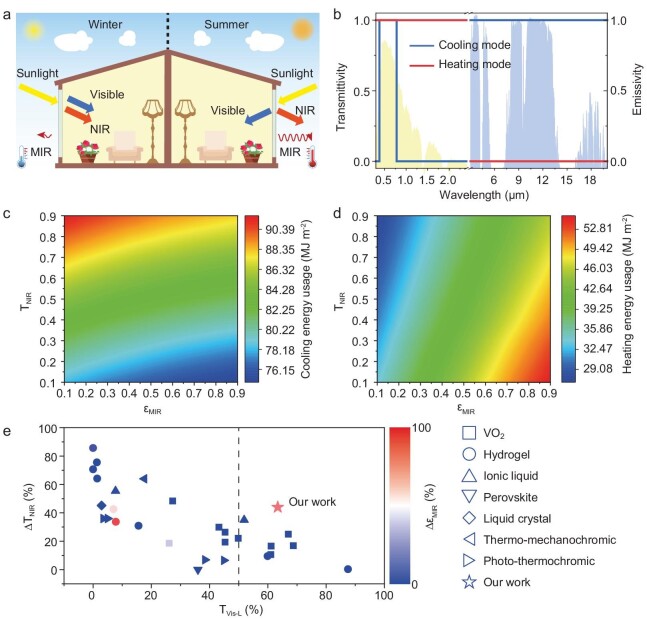
Concept and performance of thermochromic smart windows. (a) Energy transfer process of ideal thermochromic windows in winter daytime (left) and summer daytime (right). In winter, both visible and NIR light are transmitted into the room while the MIR radiation is suppressed to reduce heat loss. In summer, visible light is transmitted and the NIR is reflected through the window with high MIR emissivity to improve cooling performance. (b) The ideal spectrum of thermochromic windows against the normalized AM1.5 global solar spectrum (yellow shadow) and atmospheric transmissivity spectrum (blue shadow). The visible transmissivity of both heating and cooling modes is high, while the NIR is blocked and the MIR is enhanced when changing from heating mode to cooling mode. (c, d) Cooling and heating energy usage in different T_NIR_ and ε_MIR_ simulated on a modeled building in Nanjing. The minimum cooling/heating energy usage is achieved by low/high T_NIR_ and high/low ε_MIR_, respectively, which agrees with the ideal spectrum of thermochromic smart windows. (e) Thermochromic performance (the lowest visible transmissivity in two states, T_Vis-L_; NIR modulation, ∆T_NIR_; MIR modulation, ∆ε_MIR_) of HVTW compared with current works. HVTW in our work can achieve excellent NIR modulation and MIR modulation simultaneously with visible transmissivity of >50% in both heating and cooling modes.

Thermal radiation can be divided into the visible band (Vis band, 0.38–0.78 μm), the near-infrared band (NIR band, 0.78–2.5 μm), and the mid-infrared band (MIR band, 2.5–20 μm), which play different roles for windows. The visible band mainly determines the transparency of windows close to human's visual sense and the near-infrared band contributes to the heating effect on a room [9]. The mid-infrared band, the main band of blackbody radiation from objects on Earth, is closely interrelated with heat radiation exchange between the window and the ambient/outer space [10–12]. Those buildings with a large window area, such as central business districts, shopping malls, residential buildings, and greenhouses, which require windows with visible transmissivity of at least 50%, are the ones not energy-efficient or having stringent temperature requirements [13–17]. Thus, the ideal energy transfer process for thermochromic smart windows, in response to the ambient temperature change, is shown in Fig. 1a and Fig. S1. Under cold conditions such as winter daytime or frigid nighttime, an ideal thermochromic window should enable all the sunlight into the room for maximal solar harvesting in the daytime and possess low MIR emissivity to decrease the net radiative power outward. Inversely, in hot conditions such as summer daytime or torrid daytime, the window should still present transparency in the visible band while simultaneously possessing high NIR reflectivity to reduce extra heat input and high MIR emissivity to improve radiative cooling performance.

Therefore, to keep the intrinsic function of windows, the ideal spectrum of thermochromic smart windows should remain high visible transparency in both heating and cooling modes (Fig. 1b), while the NIR is blocked and the MIR is enhanced so as to achieve minimal net energy input when changing from heating mode to cooling mode. To clarify the relationship between building energy usage and NIR transmissivity (T_NIR_)/MIR emissivity (ε_MIR_), we conducted an energy consumption simulation of a typically commercial 3-layer medium office, following a commonplace standard (ASHRAE, Notes S1 and S2, Fig. S2 and Table S1) [13]. As shown in Fig. 1c and d, the low NIR transmissivity (T_NIR_) and high MIR emissivity (ε_MIR_) brought minimal cooling energy usage while high T_NIR_ and the low ε_MIR_ brought minimal heating energy usage with visible transmissivity consistently fixed at 0.9. It further confirms that the modulation range of NIR transmissivity and MIR emissivity will directly influence building energy usage, which agrees with the importance of the ideal spectrum mentioned above.

In past years, the modulation performance of thermochromic smart windows in different wavebands has been widely studied based on different material characteristics. In this context, vanadium dioxide (VO_2_) and hydrogel are the main materials used in thermochromic smart windows [5,6,8]. VO_2_, which has a semi-conducting state at low temperatures and a metallic state at high temperatures, can regulate NIR transmissivity independently, where its NIR modulation is inversely proportional to the visible transmissivity [18–27]. As another material commonly used in thermochromic smart windows, hydrogel exhibits excellent performance on wide spectral regulation of the sunlight band due to its reversible phase separation [28–34]. There are also some other types of thermochromic windows based on ionic liquid [35,36], perovskite [37], liquid crystal [38], and multifunctional thermochromic devices [39–42]. However, it is difficult for them to achieve large modulation of T_NIR_ while also maintaining high T_Vis_ in response to temperature change, not to mention modulating ε_MIR_. Recently, a few researchers have also emphasized the importance of the modulation of ε_MIR_ to achieve further energy savings [43,44]. The Long group first introduced MIR modulation into smart windows but they relied on independent devices to regulate MIR or NIR, respectively [43]. While Huang’s group achieved the MIR and NIR modulation simultaneously whereas the device became opaque at high temperatures [44]. Therefore, developing thermochromic windows, that are capable of keeping high visible transmissivity (T_Vis_) with high NIR modulation (∆T_NIR_) and high MIR modulation (∆ε_MIR_) in response to temperature change, is the ‘Holy Grail’ but still remains a challenge. Inspired by Lendlein's work that a temperature-memory polymer actuator was used in the heat engines with adjustable rotation rate to provide programmable window shade [45], materials possessing reversible shape change such as self-rolling VO_2_ nanomembranes and shape memory polymers can be directly used on light regulation like autonomous window blinds [20,46,47], which provide a new avenue for thermochromic smart window devices with broadband optical regulation.

Here, we develop a highly visible-transparent thermochromic window (HVTW) with broadband infrared modulation based on a two-way shape memory polymer, which shows an excellent modulation ability from visible, NIR to MIR bands. It exhibits high visible transmissivity at both high (T_Vis-h_ = 63.4%) and low (T_Vis-l_ = 86.9%) temperatures, both higher than 50%, which meets the general standard for windows in architecture [13–17]. On this basis, the HVTW achieves superior optical performance on NIR modulation (∆T_NIR_ = 44.0%) and MIR modulation (∆ε_MIR_ = 76.5%) simultaneously, compared to the current thermochromic smart windows reported (Fig. 1e and Table S2). The excellent broadband regulation property brings a temperature decrease of ∼4°C in summer daytime and achieves a temperature increase of ∼2°C in winter daytime in comparison with silica glass (SiO_2_). Also, HVTW has a temperature ∼1°C higher than silica glass in spring nighttime. It is calculated that when applying this designed thermochromic smart window as an alternative to commercial glass, the building will harvest more energy saving for each season, further achieving an average annual energy saving of 10–50 MJ m^−2^ across the world.

## RESULTS

### Working mechanism and performance characterization

**Figure 2.**
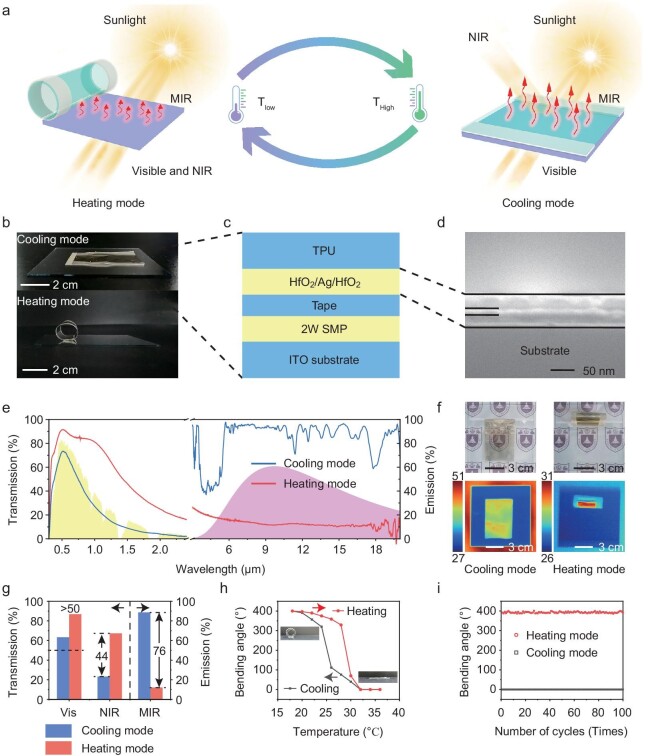
Structure, optical performance, and thermal performance of the HVTW. (a) Schematic illustration of the HVTW on the cooling and heating mode switching with temperature change. (b) Optical photographs of the HVTW on the cooling and heating modes, respectively. (c) A schematic of the designed sandwiched structure. It is composed of a TPU radiation layer with a one-dimensional photonic crystal, an adhesion tape layer, a 2W SMP layer, and an ITO substrate. (d) Cross-section scanning electron microscopic image of the one-dimensional photonic crystal. (e) Measured optical spectrum of the HVTW on the cooling (blue line) and heating (red line) mode against the normalized AM1.5 global solar spectrum (yellow shadow) and blackbody radiation spectrum (pink shadow), respectively. (f) Optical and infrared photographs on the cooling and heating modes, respectively. The unit of the scale bar in infrared photographs is °C. The optical photographs show both high visible transmissivity while the infrared photographs show high MIR emissivity on the cooling mode and low MIR emissivity on the heating mode. The red color in the infrared image on the heating mode is caused by the reflection of thermal radiation from the human body. (g) Transmissivity of the visible and NIR band and emissivity of the MIR band on the cooling (blue column) and heating (red column) mode, respectively, based on the optical spectrum in (e). (h) Bending angle of the HVTW as a function of temperature during a heating and cooling process. The illustrations show the coiled state at 20°C and the completely flat state at 32°C, respectively. (i) Bending angle of the HVTW as a function of the number of cycles on the heating and cooling process. It remains unchanged over 100 times.

The HVTW can achieve a coiled state (defined as a heating mode) and a completely flat state (defined as a cooling mode) reversibly in response to ambient temperature as shown in Fig. 2a. At low temperatures for the heating mode, HVTWs exhibit high visible and NIR transmissivity to harvest solar energy into heat and present low MIR emissivity to minimize heat loss. When the ambient temperature becomes high, HVTW automatically switches to cooling mode, which allows visible light to transmit and blocks NIR light with enhancing thermal radiation, thereby achieving minimum energy input. The optical photographs of the HVTW show a huge visual difference between cooling mode and heating mode (Fig. 2b). It consists of a thermoplastic polyurethane (TPU) radiation layer with a one-dimensional photonic crystal, an adhesion tape layer, a two-way shape memory polymer (2W SMP) layer and an indium tin oxide (ITO) substrate, to enable the broadband regulation and reversible switching process (Fig. 2c).

First, the key material is the 2W SMP as an actuating layer to realize the dynamic switching mechanism in response to temperature. A block polymer, which is composed of alternately hard (hexamethylene diisocyanate, HDI) and soft (polytetrahydrofuran, PTHF; polycaprolactone, PCL) segments (Figs S3–S5), was programmed for the special macroscopic properties of reversible heat contraction and cold expansion in length (Fig. S6). The thermodynamic property may be due to its reversible melting-crystallization process of soft segments during thermocycling (Fig. S7). Herein, PTHF functions as a spring to provide the built-in force needed to orient the recrystalline segments in the longitudinal direction and PCL acts as the switch to control the shrinkage and elongation of the 2W SMP, while HDI is considered as net-points to determine the permanent shape and mechanical strength of polymers [48]. Thus, when the adhesion tape and the TPU stick to the 2W SMP at high temperatures, the 2W SMP and adhesion tape keep the same length. When the temperature drops, the length of 2W SMP becomes larger and the length of the adhesion tape remains unchanged. For the sake of eliminating the internal force mismatch between two interfaces caused by different lengths, 2W SMP will coil together with the materials above (Fig. S8) [49]. Then, the ‘spring-switch’ composition and laminate structure ensure the tunability of the transformation temperature to meet the requirements of different practical scenarios. It can be adjusted by the treating process, especially the process when adhesion tape is attached to the 2W SMP, not merely by adjusting the molecular weight of PCL monomers. The transformation temperature is equal to the temperature when conducting the attaching process.

Second, it is essential to achieve a tuning between optical properties and mechanical properties of the device. The optical properties changing from cooling mode to heating mode require that the layer above 2W SMP should have high visible transmissivity, low NIR transmissivity and high MIR emissivity, while the mechanical properties require that it should be thin and soft so as to achieve at least one week of curling along with 2W SMP in heating mode. There is a tradeoff in thickness because the emissivity increases with the upper layer thickening while the curling performance decreases. Hence, a TPU film of 20 μm with an optimized one-dimensional photonic crystal composed of 26 nm HfO_2_/14 nm Ag/29 nm HfO_2_ (Fig. 2d) by particle swarm algorithm (Note S3) was designed as the upper layer to selectively transmit visible light but reflect NIR light (Fig. S9a) while it simultaneously acted as an excellent radiation layer with high emissivity on the whole MIR band due to its various chemical bonds such as C−O−C, C=O, and C-N (Fig. S9b). Additionally, the optical properties require that the layer below 2W SMP should have high visible transmissivity, high NIR transmissivity, and low MIR emissivity for the heating mode. Thus, the ITO substrate was chosen as the underlying layer which has low MIR emissivity and remains high transmissivity in the solar spectrum (Fig. S10). Then, to make the TPU film and 2W SMP connect tightly, a transparent optical tape of 10 μm which has a high transmissivity of >90% in the solar spectrum (Fig. S11) was selected for reducing interference to the optical performance of the device. Finally, the mechanical properties also request the thickness of 2W SMP to be lower than 100 μm so that it can drive the upper layer to achieve at least one week of curling (Fig. S12). Based on the materials above, our device can achieve high visible transmissivity, high NIR modulation, and high MIR modulation reversibly at the same time.

Third, it is also significant to tune the optical transmissivity and visual sense in the device; 2W SMP has a haze problem that will influence the visual sense although it has a negligible effect on solar transmissivity (Fig. S13). It is difficult to solve this problem because the programming process causes the 2W SMP’s many crystalline regions to change length during the melting-crystallization process which is closely related to the haze of materials. Thus, two 2W SMP stripes were arranged at intervals of 2:5 (the width of 2W SMPs: the width of our device/TPU layer above) to coil together with the entire TPU layer for device optimization (Fig. S14). This structure not only retains the optical transmissivity and the dynamic transformation process but also weakens the disadvantage in a visual sense.

With the carefully designed sandwiched structure, the optical spectrum of the HVTW on the cooling and heating mode is shown in Fig. 2e, which agrees well with the proposed ideal spectrum of thermochromic windows in Fig. 1b. The optical photographs demonstrate the high visible transmissivity on both cooling and heating mode while the infrared photographs show high MIR emissivity on the cooling mode and low MIR emissivity on the heating mode (Fig. 2f). These photographs correspond to the spectral property in Fig. 2e. Next, we carried out energy calculation based on the optical spectrum of HVTW. The results in Fig. 2g show that the visible transmissivity is 63.4% in the cooling mode and 86.9% in the heating mode which is both more than 50%. The NIR transmissivity is 23.3% and 67.3%, and the MIR emissivity is 88.7% and 12.3%, respectively, on the cooling and heating modes so that the NIR and MIR modulation can reach up to 44.0% and 76.5%, respectively, both of which exhibit excellent broadband regulation performance compared with that of other works under the condition of T_Vis_ >50%.

In addition to excellent optical performance, thermal performance such as transformation temperature and stability is comparable with other works, suitable for normal life. As shown in Fig. 2h, the coiled state of the HVTW unfolds with rising temperature and eventually keeps a completely flat state above 32°C (Movie S1). The bending angle which is defined as the angle that tangents at the end of the polymer changes from the completely flat state (0°) varies slowly at the start of the heating process and decreases sharply close to the transformation temperature. The cooling process exhibits the opposite trend and the HVTW retains a coiled state below 20°C (Movie S2). The transformation temperature of the HVTW can be adjusted by the treating process, especially the process when adhesion tape is attached to the 2W SMP (Fig. S8). For example, the device was designed with a coiled state below 30°C and a completely flat state above 42°C to cope with hotter environments (Fig. S15). Then as shown in Fig. 2i, the reversible transformation performance of the HVTW remains unchanged during repeated heating and cooling over 100 times, which demonstrates the excellent stability of our device toward long-term use. It is also verified to be effective after 3 months (Fig. S16). Therefore, all the results above suggest that our designed HVTW can achieve high NIR and MIR modulation synchronously while keeping high visible transmissivity and exhibiting outstanding thermal transformation performance and stability.

### Temperature regulation tests

**Figure 3.**
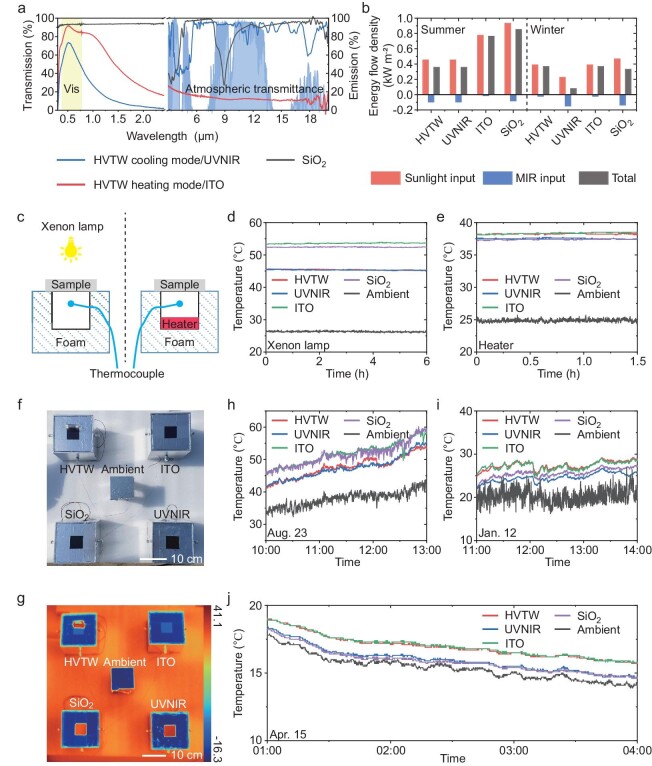
Temperature regulation performance. (a) The optical spectra of the HVTW, the UVNIR sample, ITO glass, and SiO_2_ glass as controls. (b) The power of sunlight input, MIR input, and total radiative energy through different controls in a simulated environment. The HVTW enables the lowest energy input in summer and the highest energy input in winter, which is expected to be beneficial in lowering building energy consumption for heating and cooling in all seasons. (c) The schematics of experimental setups indoors to verify the cooling performance under a xenon lamp (left) and insulation performance with a heater in the chamber (right) corresponding to different environments. (d) Temperature comparisons of different controls under a xenon lamp. (e) Temperature comparisons of different controls with a heater in the chamber. (f, g) The optical and infrared photographs of experimental setups outdoors, respectively. The unit of the scale bar in the infrared photograph is °C. (h) Temperature comparisons in summer daytime. The HVTW has the best cooling performance. (i) Temperature comparisons in winter daytime. The HVTW has the best heating performance. (j) Temperature comparisons in spring frigid nighttime. The HVTW has the best insulation performance.

The temperature regulation effect caused by the excellent optical performance of the HVTW is then evaluated via indoor and outdoor tests. A UVNIR sample consisting of an ITO glass and the TPU layer with a one-dimensional optical crystal, an ITO glass, and a SiO_2_ glass were set as controls (Fig. S17). The corresponding spectrum of these control groups is presented in Fig. 3a. The UVNIR sample and the ITO glass exhibit the same optical performance as the HVTW in the cooling and heating modes, respectively. However, they are just suitable for specific season energy saving and have negative effects in other seasons, while the HVTW has all-season energy conservation due to its automatic transformation property in response to temperature. The SiO_2_ glass is the most commonly used as a comparison having high solar transmissivity and high MIR emissivity.

Based on the optical spectrum of control groups in Fig. 3a, radiative energy analysis was carried out in a simulated environment (see Note S4 for more details). It is assumed that the sunlight power is 1000 W m^−2^ and the ambient temperature is 35°C in summer daytime while the sunlight power is 500 W m^−2^ and the ambient temperature is 15°C in winter daytime with the room temperature of 25°C. The energy flow density in Fig. 3b demonstrates that the HVTW enables the lowest energy input in summer and the highest energy input in winter compared to other controls, which is beneficial to the all-season energy-saving effect. In detail, the HVTW and the UVNIR have the lowest sunlight input due to blocking NIR (452.2, 452.2, 775.6 and 931.6 W m^−2^ for the HVTW, the UVNIR sample, ITO glass and SiO_2_ glass, respectively) and have the lowest MIR radiation input (−95.9, −95.9, −13.0 and −81.3 W m^−2^ for the HVTW, the UVNIR sample, ITO glass and SiO_2_ glass, respectively) in summer. Thus, the HVTW and the UVNIR can lower total energy load by 406.3 and 494.0 W m^−2^ in comparison with ITO glass and SiO_2_ glass. However, during winter daytime, the UVNIR retains the lowest sunlight input (387.8, 226.1, 387.8 and 465.8 W m^−2^ for the HVTW, the UVNIR sample, ITO glass and SiO_2_ glass, respectively) and lowest MIR radiation input (−20.8, −148.2, −20.8 and −135.7 W m^−2^ for the HVTW, the UVNIR sample, ITO glass and SiO_2_ glass, respectively). It will increase the total energy load for heating by 281.9, 281.9 and 252.2 W m^−2^ in comparison with the HVTW, ITO glass and SiO_2_ glass, respectively, which is disadvantageous to energy saving in winter. Hence, the HVTW exhibits highly efficient energy conservation all year round.

To further verify that HVTW is capable of regulating temperature effectively, an indoor simulation experiment was designed with the setup chamber size of 5 cm × 5 cm × 6 cm schematically shown in Fig. 3c. The setup was insulated from heating conduction by the foam, and the thermocouple probe was located in the center of the chamber. Under xenon lamp radiation with an intensity of 1.4 kW m^−2^, the HVTW was in a completely flat state and exhibited a similar temperature to the UVNIR sample, which could achieve a cooling temperature of 8°C and 7°C compared with ITO glass and SiO_2_ glass, respectively (Fig. 3d). It clearly proved that our HVTW has a better cooling effect in a hot environment. Then, an experiment was carried out with a heater in the chamber to verify the thermal insulation performance of the HVTW. As shown in Fig. 3e, the HVTW presented similar performance with ITO glass due to its low MIR emissivity in a coiled state, which could achieve a temperature increase of 1°C compared with the UVNIR sample and SiO_2_ glass. Therefore, the HVTW can effectively regulate chamber temperature in response to changes in the environmental temperature.

We further measured temperature with the setups in Fig. 3f outdoors. Four different types of windows (the HVTW, the UVNIR sample, ITO glass and SiO_2_ glass with the same dimension of 5 cm × 5 cm × 1.1 mm) were equipped on the chamber with a size of 15 cm × 5 cm × 6 cm. The ambient temperature thermocouple was located under an Al foil covering to avoid the influence of sunlight. The corresponding infrared photographs demonstrated that the HVTW at a coiled state and ITO glass exhibit low MIR emissivity while the UVNIR sample and SiO_2_ glass have high MIR emissivity which agrees with the optical spectrum in Fig. 3a (Fig. 3g). Due to the strong sunlight intensity and high ambient temperature in summer daytime, our HVTW was at a completely flat state so that its chamber temperature was consistent with the UVNIR and 4°C cooler than that under ITO glass and SiO_2_ glass (Fig. 3h and Fig. S18a). While in winter daytime, the ambient temperature was low and our HVTW was in a coiled state. Consequently, the HVTW exhibited the highest temperature when compared to ITO glass, which had a temperature increase of 3°C and 2°C in comparison with the UVNIR and SiO_2_ glass (Fig. 3i and Fig. S18b). Meanwhile, in spring frigid nighttime, our HVTW at a coiled state also had a temperature increase of 1°C compared to the UVNIR sample and SiO_2_ glass (Fig. 3j and Fig. S19). Considering that there was no external energy input from the sun, thermal radiation played a dominant role in chamber temperature. Hence, our HVTW and ITO glass exhibited higher chamber temperature due to its low MIR emissivity. These temperature results above are all consistent with our expectations in energy analysis. All the results verify that the designed HVTW possesses excellent temperature regulation capabilities in various environments, which is essential for building energy saving in all seasons.

### Real-time thermal regulation and energy-saving simulation

**Figure 4.**
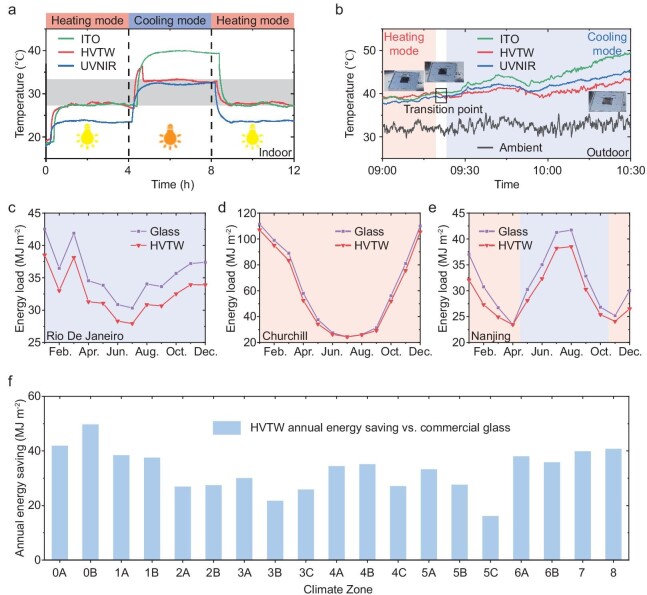
Real-time thermal regulation and energy simulation. (a) The temperature transformation under simulated season by changing the xenon lamp intensity and ambient temperature. The bulb with light color represents simulated winter with a xenon lamp intensity of 500 W m^−2^ and an ambient temperature of 20°C while the bulb with dark color represents simulated summer with a xenon lamp intensity of 1000 W m^−2^ and an ambient temperature of 34°C. Our HVTW can complete a whole temperature transformation process. (b) The temperature change of the HVTW outdoors. The inserts describe different states of the HVTW as time goes by. Our HVTW can change from heating mode (red shadow) to cooling mode (blue shadow) due to the rising sunlight intensity and ambient temperature. (c–e) Monthly energy load of the HVTW and commercial glass in Rio De Janeiro, Churchill and Nanjing, respectively. Our HVTW exhibits an energy saving effect quarterly in all typical cities. (f) Average annual energy saving with the HVTW in 19 climate zones globally against a commercial glass as the baseline. Our HVTW achieves better performance than glass anywhere in the world.

We further carried out real-time thermal regulation tests for HVTW to record the continuous variation process. As shown in Fig. 4a, we observed the chamber temperature transformation directly caused by the thermal regulation of the HVTW under changing xenon lamp intensity and ambient temperature to simulate seasonal variation (Fig. S20). In simulated winter with a light intensity of 500 W m^−2^ and an ambient temperature of 20°C, our HVTW was in a coiled state so that it exhibited higher temperature the same as ITO glass. When the local environment was changed to simulated summer with a light intensity of 1000 W m^−2^ and an ambient temperature of 34°C, the HVTW unfolded gradually until it was in a completely flat state. Thus, the HVTW on the cooling mode had a lower temperature than ITO glass and enabled a similar temperature to the UVNIR. As expected, when changing the environment to simulated winter again, the temperature of the HVTW was still as high as ITO glass which indicated that the HVTW had returned to heating mode. Therefore, our HVTW can spontaneously complete the whole transformation process in changing circumstances and ambient temperatures.

Then, we recorded the transformation process by real-time thermal test outdoors. Our HVTW was at a coiled state initially due to the low temperature in the morning and consequently enabled higher temperature similar to ITO glass (Fig. 4b). As time went by, sunlight intensity and ambient temperature rose (Fig. S21), which contributed to the unfolding process of the HVTW marked as ‘transition point’ as shown in Fig. 4b. Thus, the HVTW was at a completely flat state corresponding to cooling mode and its chamber temperature was cooler than that of ITO glass. This suggests that the transformation process of our HVTW works just as well outdoors.

As the excellent thermal regulation performance has been proven, the HVTW is expected to achieve building energy savings for all seasons globally. A medium office model in Energyplus software was used to simulate the impacts on the monthly and annual energy consumption when applying the HVTW in comparison with commercial glass (Note S5, Fig. S2 and Table S3). There are 19 climate zones divided in ANSI/ASHRAE Standard and we first selected three typical cities in different climate zones as representation [50,51]. They were Rio De Janeiro (Zone 1A) with year-round hot weather, Churchill (Zone 8) with year-round cold weather, and Nanjing (Zone 3A) with four seasons, respectively (Fig. S22). In Rio De Janeiro, our HVTW remained in the cooling mode throughout the year and therefore withstood less energy load than commercial glass in each month (Fig. 4c). In Churchill, our HVTW kept the heating mode all year round so that its insulation effect brought less energy load per month as well (Fig. 4d). In Nanjing with four seasons, the HVTW was in cooling mode from May to October while switching to heating mode in other months, which still exhibited less or considerable energy load than commercial glass (Fig. 4e). Therefore, it was verified from three representative cities that our HVTW can enable building energy saving for all seasons superior to commercial glass. Then, we calculated the annual building energy consumption with HVTW and commercial glass in 19 typical cities in different climate zones as representation (Figs S23 and S24, and Table S4). As shown in Fig. 4f, our HVTW could achieve an annual energy saving of 10–50 MJ m^−2^ in comparison with commercial glass anywhere in the world. Additionally, due to changing monthly average temperatures in different regions, the optimal transformation temperature of the HVTW can be determined and set for each climate zone to achieve maximum local energy savings (Note S5 and Fig. S25). Finally, the potential on the scalability of the HVTW is discussed for practical application (Note S6 and Figs S26–S28).

## DISCUSSION

In summary, we propose and demonstrate a highly visible-transparent thermochromic smart window with excellent optical modulation properties that exhibit NIR modulation of 44.0% and MIR modulation of 76.5% simultaneously while retaining visible transmissivity of more than 50.0% for both cooling and heating modes. It benefits from the ‘spring-switch’ design and laminate structure together exhibiting a two-way shape memory effect, the tuning between optical properties and mechanical properties, and the tuning between optical transmissivity and visual sense in the device. The optical modulation can directly lead to thermal regulation, which verifies that the HVTW can achieve lower indoor temperature in summer daytime and higher indoor temperature in both winter daytime and spring nighttime. Then a whole transformation process of the HVTW with chamber temperature regulation is recorded to describe the feasibility of our device in perceiving temperature. It is expected that this designed HVTW is compatible with conventional electrochromic smart windows which can actively regulate visible transparency and shows the potential to pave the way for thermochromic smart windows for energy-efficient buildings towards global net-zero carbon.

## Supplementary Material

nwae408_Supplemental_Files
