# Supplementary material for: A highly visible-transparent thermochromic smart window with broadband infrared modulation for all-season energy savings
Source: Natl Sci Rev. 2024 Nov 13;12(2):nwae408. doi: 10.1093/nsr/nwae408 (PMC11737393; doi:10.1093/nsr/nwae408)
Supplement: nwae408_Supplemental_Files [file nwae408_supplemental_files.zip › Supplementary data.pdf]

Supporting Information

A highly visible-transparent thermochromic smart window with broadband infrared modulation for all-season energy savings

Yi Jiang[1]†, Yunlong Wang[1]†, Deshuo Kong[2]†, Zipeng Chen[1], Zhengwei Yang[1], Ningning Cao[1], Haowen Chi[1], Shining Zhu[1], Qiuhong Zhang[2]*, Jia Zhu[1]*, Bin Zhu[1]*

[1] National Laboratory of Solid State Microstructures, College of Engineering and Applied Sciences, Jiangsu Key Laboratory of Artificial Functional Materials, Frontiers Science Center for Critical Earth Material Cycling, Collaborative Innovation Center of Advanced Microstructures, Nanjing University, Nanjing 210093, P. R. China.

[2] Key Laboratory of High Performance Polymer, Material and Technology of MOE, Department of Polymer Science and Engineering, School of Chemistry and Chemical Engineering, Nanjing University, Nanjing 210093, P. R. China.

†These authors contributed equally to this work

*E-mail: chemzqh@nju.edu.cn; jiazhu@nju.edu.cn; binzhu@nju.edu.cn;

Materials and Methods

Fabrication of the two-way shape memory polymer

The two-way shape memory polymer was fabricated by one-step synthesis. Firstly, dry polytetrahydrofuran (PTHF, number-average molecular weight = 1000, Aladdin) and polycaprolactone (PCL, number-average molecular weight = 36000~45000, Aladdin) were fully stirred for dissolving in trichloromethane ($CHCl_3$, 99.5%, Yonghua Chemical Co., Ltd) in proportion at room temperature. Then, the solution was added by hexamethylene diisocyanate (HDI, 99%, Aladdin) and dibutyltin dilaurate (DBTBL, AI LAN Chemical Technology Ltd) in sequence and continued to stir for 12 h at room temperature. The molar ratio of three monomers (n_{PTHF}, n_{PCL}, n_{HDI}) was 25: 3: 50. The amount of DBTBL catalyst was 0.7% of the total weight of the three monomers. After the reaction, the 2W SMP product was poured into a hollow mold made of polytetrafluoroethylene. After removing the bubbles from the reaction mixture for a while in the refrigerator, the solvent was allowed to evaporate at room temperature to produce the film. The 2W SMP film could be peeled off the mold after complete drying and cut into different shapes for further usage.

Fabrication of the highly visible-transparent thermochromic smart window with broadband infrared modulation

The device in Fig. 2 is chosen as an example. An as-prepared 2W SMP stripe was stretched out to five times its initial length at 90℃ and fixed until cooling to room temperature (~ 20℃). The stretched 2W SMP stripe retained the even middle section after being cut off both ends and then shrunk naturally along the stretching direction at 32℃. A piece of same-size optical tape was attached to the 2W SMP stripe at 32℃. Two 2W SMP stripes with optical tape attached were arranged side by side at intervals of 2:3 (the width of 2W SMPs: the distance between stripes) at 32℃. Then, a TPU layer, which was deposited with the one-dimensional photonic crystal above consisting of 26 nm HfO_2/ 14 nm Ag/ 29 nm HfO_2 in order by magnetron sputtering, was covered on two 2W SMP stripes to form a significantly sized film at 32℃. The ratio of the width of the TPU layer to 2W SMP was 5:2 and the length of both the TPU layer and 2W SMP was the same. Finally, this film was fixed on an ITO glass by a piece of narrow tape, which became an integrated highly visible-transparent thermochromic smart window with broadband infrared modulation. This device could be in a coiled state at room temperature (~ 20℃).

Material characterizations

The cross-sectional microscopic image of the one-dimensional photonic crystal was captured by scanning electron microscopy (SEM; MIRA3, TESCAN). The transmissivity of samples in the solar spectrum (0.3-2.5 μm) was measured using ultraviolet-visible spectroscopy (UV3600, SHIMADZU) equipped with an integrating sphere (ISR-310). The emissivity of samples in the mid-infrared band (2.5-20 μm) was measured using a Fourier transform infrared spectrophotometer (Nicolet IS50R, Thermo Fisher Scientific) loaded with an integrating sphere (4P-GPS-020-SL, Pike). The infrared photographs were captured by an infrared camera (TiX580, Fluke).

Indoor temperature tests

The temperature was monitored by K-type thermocouples and real-time recorded by a recorder (MIK R6000C, Asmik). A xenon lamp (Solar-500, Yingwave Optics) with an optical filter for the standard AM1.5 spectrum was used as the illuminant. The light intensity was calibrated by a thermopile power sensor (GCI-080250, Daheng Optics). A Kapton heater sheet was used as an internal heat source with the size of 5 cm × 5 cm.

Outdoor temperature tests

The temperature was monitored through the same method used in the indoor test in Nanjing (32° 3′ 18.8″ N, 188° 46′ 33.2″ E). The power of incident sunlight was recorded by a solar radiation meter (TBQ).

Notes

Note S1. The theoretical formula of optical properties

The visible transmissivity (T_{Vis}) and near-infrared transmissivity (T_{NIR}) can be expressed as follows, respectively:

$$T_{Vis} = \int_{0.38}^{0.78} T(\lambda) I_{AM1.5}(\lambda) d\lambda \Big/ \int_{0.38}^{0.78} I_{AM1.5}(\lambda) d\lambda$$

$$T_{NIR} = \int_{0.78}^{2.5} T(\lambda) I_{AM1.5}(\lambda) d\lambda \Big/ \int_{0.78}^{2.5} I_{AM1.5}(\lambda) d\lambda$$

where, $T(\lambda)$ is transmissivity as a function of wavelength (μm), $I_{AM1.5}(\lambda)$ is the reference direct normal solar spectral irradiance ASTM G173-03 under air-mass 1.5.

The mid-infrared emissivity (ε_{MIR}) can be expressed as follows:

$$\varepsilon_{MIR} = \int_{2.5}^{20} \varepsilon(\lambda) I_{BB}(\lambda) d\lambda \Big/ \int_{2.5}^{20} I_{BB}(\lambda) d\lambda$$

where, $\varepsilon(\lambda)$ is emissivity as a function of wavelength (μm), $I_{BB}(\lambda)$ is the spectral irradiance of a blackbody at a certain temperature.

The modulation performance of near-infrared transmissivity (ΔT_{NIR}) can be expressed as follows:

$$\Delta T_{NIR} = T_{NIR-H} - T_{NIR-C}$$

where, T_{NIR-H} is the near-infrared transmissivity on the heating mode and T_{NIR-C} is the near-infrared transmissivity on the cooling mode.

The modulation performance of mid-infrared emissivity ($\Delta\varepsilon_{MIR}$) can be expressed as follows:

$$\Delta\varepsilon_{MIR} = \varepsilon_{MIR-C} - \varepsilon_{MIR-H}$$

where, ε_{MIR-H} is the mid-infrared emissivity on the heating mode and ε_{MIR-C} is the mid-infrared emissivity on the cooling mode.

Note S2. Cooling/heating energy usage simulation vs T_{NIR} and ε_{MIR}

A full-scale building with the model of a typically commercial 3-layer medium office with a floor dimension of 49.9 m (163.8 ft) × 33.3 m (109.2 ft) × 4.0 m (13 ft) was proposed to verify that building cooling /heating energy usage is closely related to NIR transmissivity (T_{NIR}) and MIR emissivity (ε_{MIR}) (Fig. S2). This model is supported by the Pacific Northwest National Laboratory (PNNL) of the U. S. Department of Energy (DOE), which is equipped with universal architecture, HVAC system, internal loads and schedules, and miscellaneous loads as a commercial building following applicable energy codes and standards [1-3]. The detailed

information on this model is shown in Table S1. Only the parameters of the window were adjusted to reveal its effect on building cooling /heating energy usage. Assuming that the visible transmissivity of the window is 0.9, a mapping of cooling /heating energy usage vs T_{NIR} (0.1-0.9) and ε_{MIR} (0.1-0.9) in Nanjing is displayed in Fig. 1c and 1d, respectively. The maximum cooling energy usage point was located at T_{NIR} = 0.9 and ε_{MIR} = 0.1, and the minimum cooling energy usage point was located at T_{NIR} = 0.1 and ε_{MIR} = 0.9. Analogously, the maximum heating energy usage point was located at T_{NIR} = 0.1 and ε_{MIR} = 0.9, and the minimum cooling energy usage point was located at T_{NIR} = 0.9 and ε_{MIR} = 0.1. Hence, ideal windows are required to achieve transformation from minimum cooling energy usage in a hot environment (T_{NIR} = 0.1, ε_{MIR} = 0.9) and minimum heating energy usage in a cold environment (T_{NIR} = 0.9, ε_{MIR} = 0.1), which agrees with the ideal spectrum in Fig. 1b.

Note S3. Design process on one-dimensional photonic crystal

The design process of one-dimensional photonic crystal mainly relies on particle swarm optimization which is a heuristic algorithm inspired by animals' behavior with a local optimization method. The details are as follows.

For a *N*-layer one-dimensional photonic crystal, the refractive index can be described as $\boldsymbol{n(\lambda)}$ = $[n_1(\lambda), \dots,\ n_N(\lambda)]^T$ and thickness can be described as d = $[d_1, \dots,\ d_N]^T$. The transmission $T(\boldsymbol{n}, \boldsymbol{d}, \lambda)$ can be calculated using the transfer matrix method. To find the optimal structure, the goal is to make theoretical transmission spectrum $T(\boldsymbol{n}, \boldsymbol{d}, \lambda)$ approach ideal transmission spectrum $T^*(\lambda)$, which can be expressed by the merit function $F(\boldsymbol{n}, \boldsymbol{d}, \lambda)$.

$$F(\boldsymbol{n}, \boldsymbol{d}, \lambda) = \sum_{\lambda} X(\lambda)\big(T(\boldsymbol{n}, \boldsymbol{d}, \lambda) - T^*(\lambda)\big)^2$$

where $X(\lambda)$ describes the energy weight of sunlight associated with each wavelength.

For photonic crystals with different structure composition, they can be treated as a list of integers that change from 1 to *M* defined as $\boldsymbol{\tilde{n}}$. To achieve the optimal spectrum, the structure design is expressed as the following formula:

$$[\boldsymbol{\tilde{n}}^*, \boldsymbol{d}^*] = \underset{\boldsymbol{\tilde{n}} \in Z^N, \boldsymbol{d} \in R^N}{\operatorname{argmin}} F(\boldsymbol{\tilde{n}}, \boldsymbol{d})$$

where $\boldsymbol{\tilde{n}}^*$ and $\boldsymbol{d}^*$ represent the optimal material combinations and layer thickness, respectively.

We firstly randomly generate *M* number of *N*-layered (*N*<5) structures with random thickness as original data. In each structure, the optimization process is as follows. The main parameters

consist of location (the spectrum of the current structure with a thickness series), velocity (vary the thickness of each layer), fitness (evaluate the spectral error between current spectrum and ideal spectrum), individual optimal solution (the optimal structure in the last iteration) and global optimal solution (the optimal structure in all iterations that have gone through). The local optimization is performed by updating the above parameters in iterations until satisfying the given minimum fitness. After many loops, we finally achieve the one-dimensional photonic crystal composed of 26 nm HfO_2/ 14 nm Ag/ 29 nm HfO_2 for the desired spectrum.

Note S4. Energy analysis of different samples in a simulated environment

The total energy analysis input of a sample is divided into the sunlight input and mid-infrared input, which can be expressed as:

$$P_{net} = P_{sun} + P_{MIR}$$

The sunlight input (P_{sun}) can be obtained by the following equation:

$$P_{sun} = \int_{0.3}^{2.5} T(\lambda) I_{AM1.5}(\lambda) d\lambda$$

where, $T(\lambda)$ is the sunlight transmissivity and $I_{AM1.5}(\lambda)$ is the reference direct normal solar spectral irradiance ASTM G173-03 under air-mass 1.5.

The mid-infrared input (P_{MIR}) can be obtained by the following equation:

$$P_{MIR} = \int_{2.5}^{20} \varepsilon_{amb}(\lambda)\varepsilon_{s}(\lambda) I_{amb}(\lambda) d\lambda - \int_{2.5}^{20} \varepsilon_{s}(\lambda) I_{s}(\lambda) d\lambda$$

where, $\varepsilon_{amb}(\lambda)$ and $\varepsilon_{s}(\lambda)$ is the emissivity of ambient air and samples as a function of wavelength (μm), respectively. $I_{amb}(\lambda)$ and $I_{s}(\lambda)$ is the spectral irradiance of a blackbody at a temperature of ambient air (T_{amb}) and samples (T_s).

The simulated summer daytime has a sunlight power of 1000 W m^{-2} and an ambient temperature of 35℃, while the simulated winter daytime has a sunlight power of 500 W m^{-2} and an ambient temperature of 15℃.

The simulated room temperature (sample temperature) is set as 25℃.

Note S5. Energy consumption monthly and annually of the HVTW in comparison with commercial glass

The same model in Note S2 was proposed for energy consumption. The optical properties of different windows used in the simulation are shown in Table S3, with other components of the

model remaining unchanged. The HVTW can change from heating mode to cooling mode when the monthly average temperature is higher than the transformation temperature, which was set as 16℃. 19 climate zones divided in ANSI/ASHRAE Standard were selected to make performance comparisons between the HVTW and commercial glass on a global scale [4,5]. The typical cities under climate zones around the world were chosen for simulation: Bangkok metropolis, Thailand (Zone 0A), Abu Dhabi Intl, United Arab Emirates (Zone 0B), Rio De Janeiro galeao, Brazil (Zone 1A), New Delhi Indira Gandhi Intl, India (Zone 1B), Hong Kong Cheung Chau, China (Zone 2A), Lima, Peru (Zone 2B), Nanjing, China (Zone 3A), San Diego, Califonia, United States (Zone 3B), Santiago Pudahuel, Chile (Zone 3C), Beijing, China (Zone 4A), Tianjin, China (Zone 4B), Pamplona, Spain (Zone 4C), Graz, Austria (Zone 5A), Klamath, Oregon, United States (Zone 5B), Port Hardy, British Columbia, Canada (Zone 5C), Helsinki Harmaja, Finland (Zone 6A), Helana, Montana, United States (Zone 6B), Edmonton Intl, Alberta, Canada (Zone 7), Churchill, Manitoba, Canada (Zone 8). The results on the monthly energy load of the HVTW in comparison with commercial glass in these representative cities are shown in Fig. 4c-e and Fig. S23-24. The HVTW could enable building energy saving for all season superior to commercial glass. Then, we calculated the annual energy saving in different climate zones with 19 typical cites as representation and achieved average annual energy saving with the HVTW globally against a commercial glass as the baseline (Table S4 and Fig. 4f). The building energy saving per unit area is based on floor area. Additionally, due to the changed monthly average temperature in different regions, the optimal transformation temperature of the HVTW can be determined and set for each climate zone to achieve the maximum local energy savings. Take Nanjing in Zone 3A for example, the cooling mode of the HVTW harvests the maximum energy savings from April to October while the heating mode harvests the maximum in other months (Fig. S25a). Thus, the optimal transformation temperature of the HVTW in Nanjing should be set within the range of monthly temperature from March to April and from September to October, which is 11.3-15.9℃ according to Fig. S25b.

Note S6. Blueprint on the scalability of the HVTW

Considering the practical application of the HVTW, we can prepare a large-area sample with array form on polyethylene terephthalate (PET) substrate deposited with ITO and attach it inside double glazing [6-8]. The double glazing spacing is decided by the coiled diameter of the HVTW, which further depends on the thermodynamic property of 2W SMP, the intervals of two 2W

SMP stripes in a device. As shown in Fig. S26-27, the HVTW inside double glazing could remain working with a spacing of 2 cm. For ulterior scalability, the fabrication and application of the HVTW can be illustrated in Fig. S28. The 2W SMP can be cut into rectangle shapes with the same size initially, which then are stretched for programming at high temperatures (such as 90℃) uniformly and fixed until cooling to room temperature (20℃). These 2W SMP stripes are placed side by side on the heating stage based on the required transformation temperature (such as 32℃) and then attached to optical tapes of the same size. The one-dimensional photonic crystal can be prepared with physical vapor deposition (PVD) such as magnetron sputtering, which is expected to be scalable via a roll-to-roll way. Then, the 2W SMP stripes and the one-dimensional photonic crystal together form a structural unit and are arranged in array mode on a flexible substrate such as polyethylene terephthalate (PET). The HVTW with an array structure can achieve transformation in response to temperature change and is convenient to be coupled with double glazing.

Figures

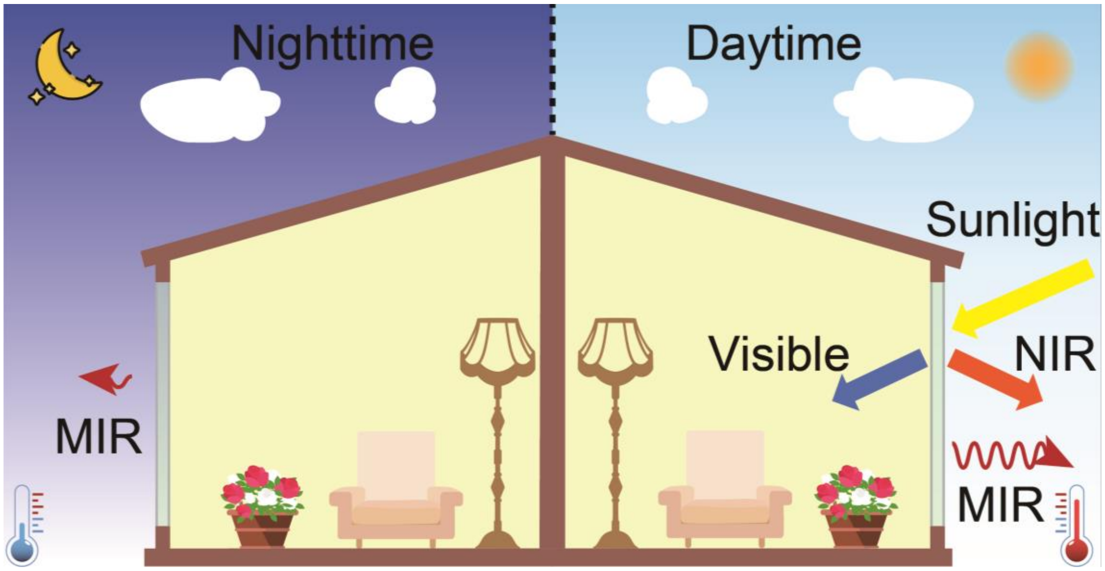

Figure. S1. Energy transfer process of ideal thermochromic windows in frigid nighttime (left) and torrid daytime (right). In the frigid nighttime, the MIR radiation is suppressed to reduce heat loss. In torrid daytime, visible light is transmitted and the NIR is reflected through the window with high MIR emissivity to improve cooling performance.

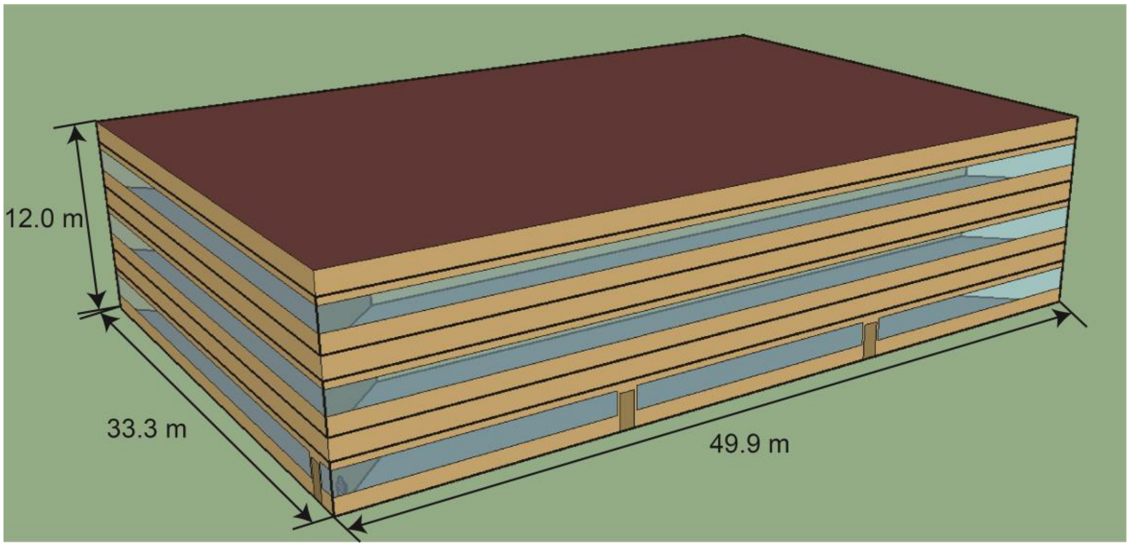

Figure. S2. Medium office building model used in an actual-size building energy consumption simulation.

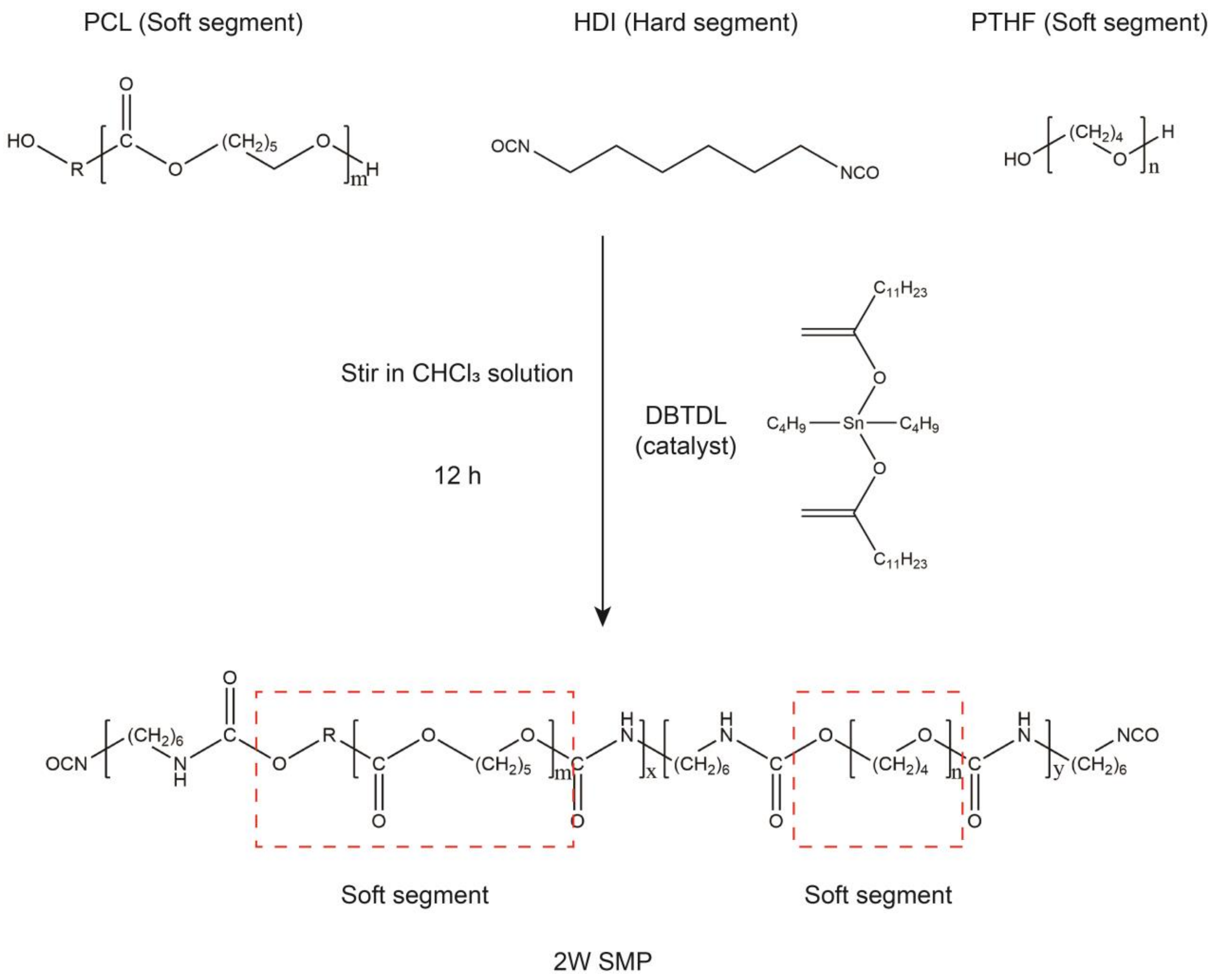

Figure. S3. Synthesis route to 2W SMP.

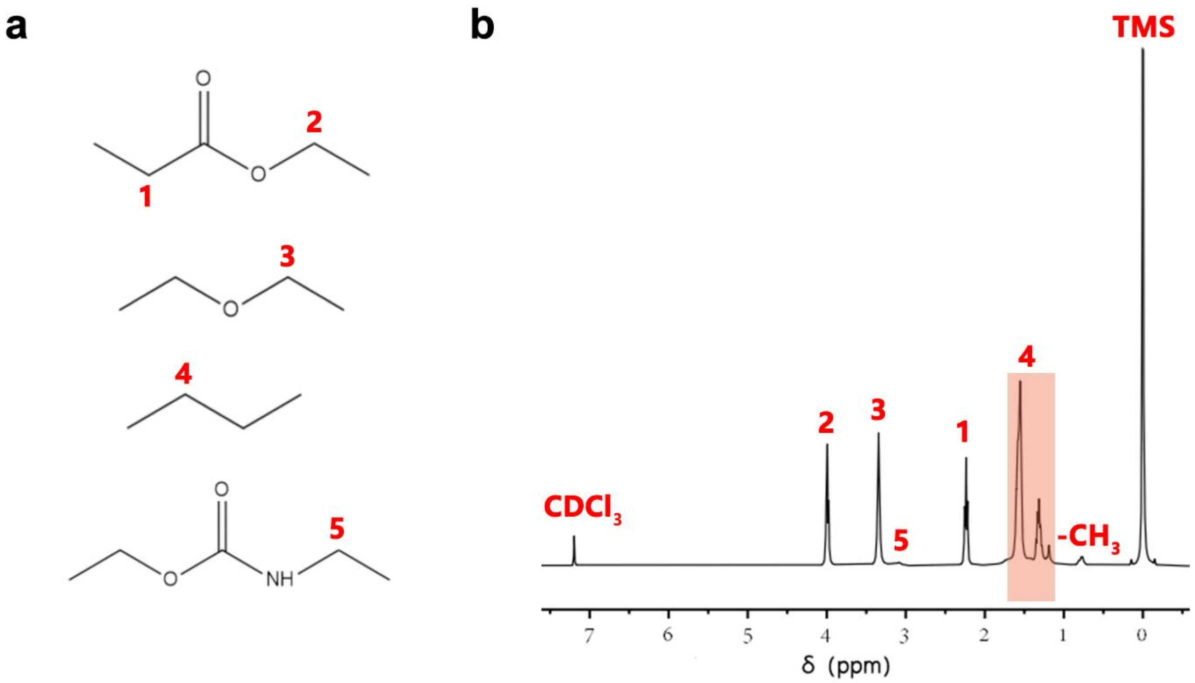

Figure. S4. Characterization of 2W SMP. (a) Characteristic molecular structures in 2W SMP. **(b)** 1H NMR spectrum of 2W SMP in $CDCl_3$ (including 0.03% TMS).

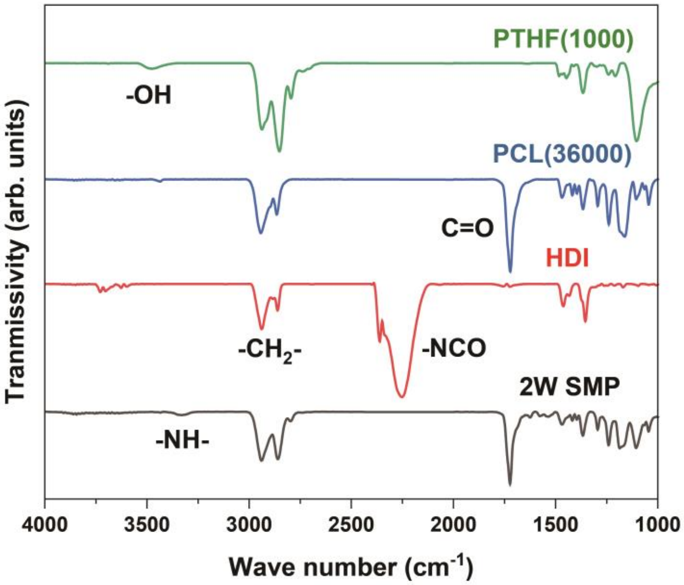

Figure. S5. FTIR transmissivity spectrums of PTHF, PCL, HDI, and 2W SMP.

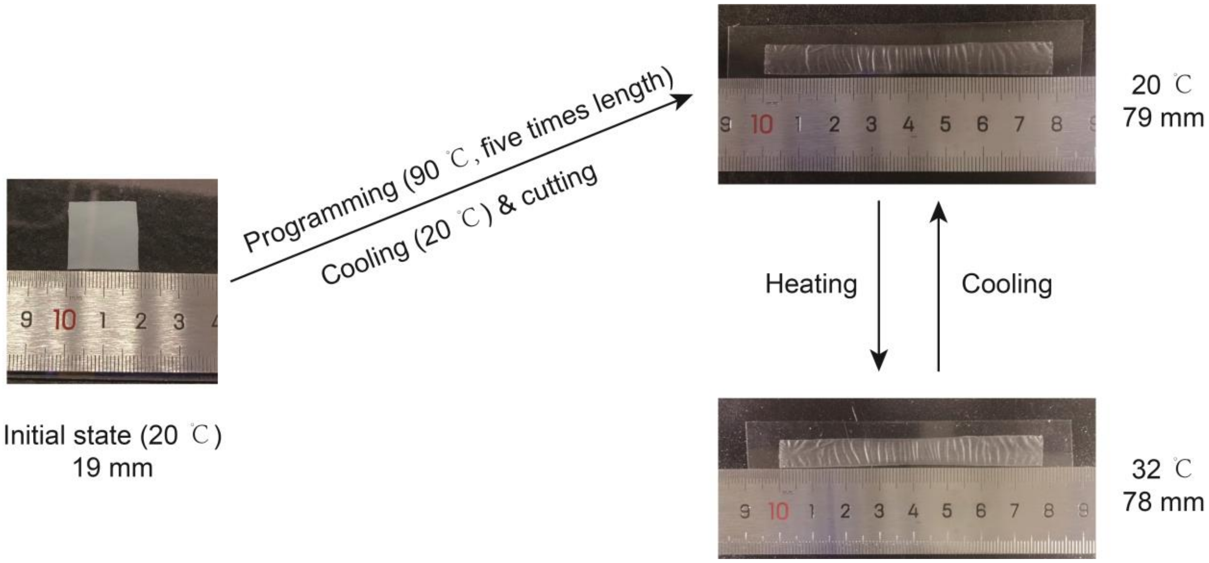

Figure. S6. The thermodynamic property of 2W SMP after the programming process. The 2W SMP stripe demonstrates the macroscopic properties of heat contraction and cold expansion due to its reversible melting-crystallization process of soft segments during thermocycling.

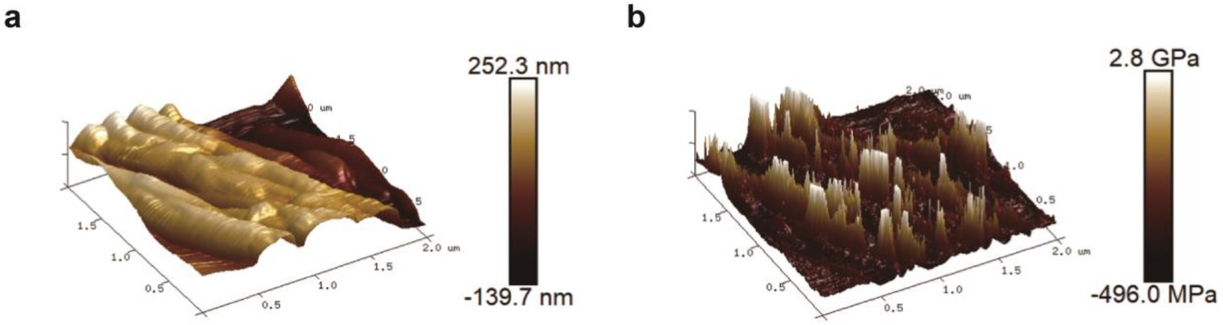

Figure. S7. (a, b) Surface profile and elastic modulus map of 2W SMP after stretched (Scan size is 2 μm). The variations in the elastic modulus suggest degrees of the orientations after stretched.

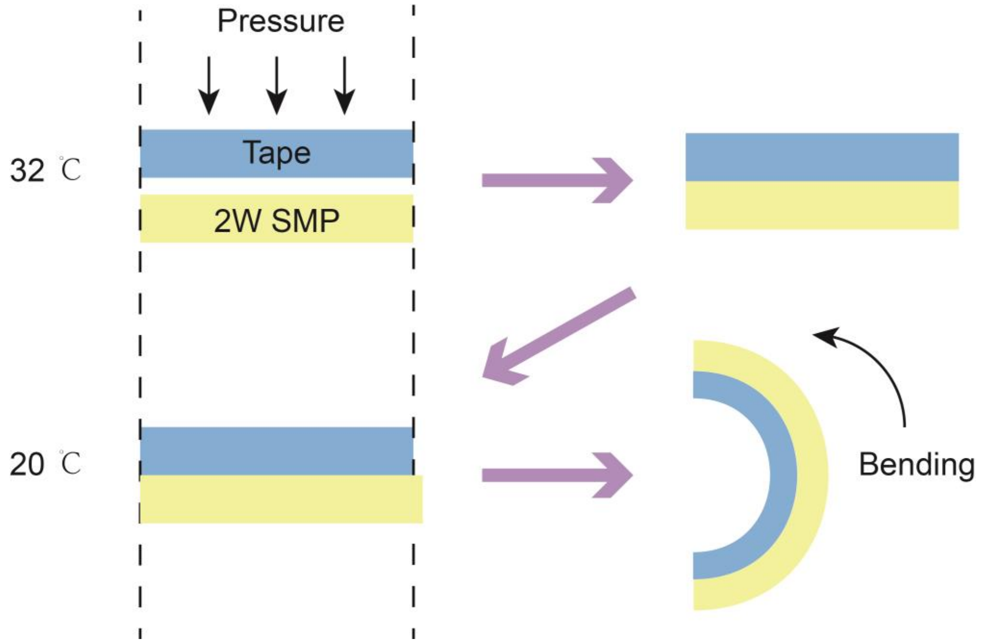

Figure. S8. The thermodynamic response behavior caused by the internal force mismatch between the interface of optical tape and 2W SMP. Take the transformation temperature which is set as 32℃ for example, when the tape is stuck to the 2W SMP at 32℃, the lengths of the adhesion tape and 2W SMP are the same. While the temperature drops to 20℃, the 2W SMP exhibits longer length but the tape remains unchanged in length, which leads to the coiled behavior of 2W SMP together with the materials above.

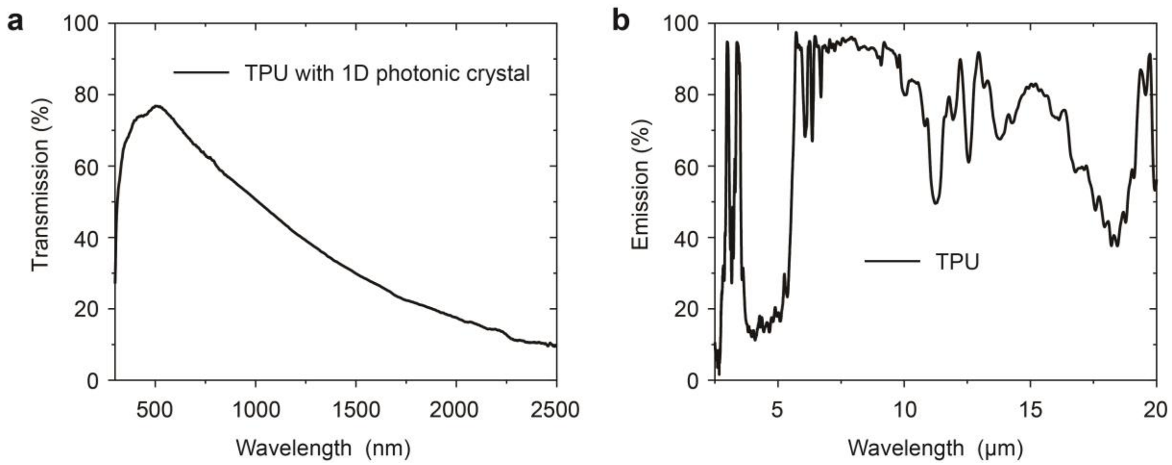

Figure. S9. The optical spectrum of the layer above 2W SMP. (a) The solar transmission spectrum of the TPU film with one-dimensional photonic crystal. **(b)** The emission spectrum of the TPU film.

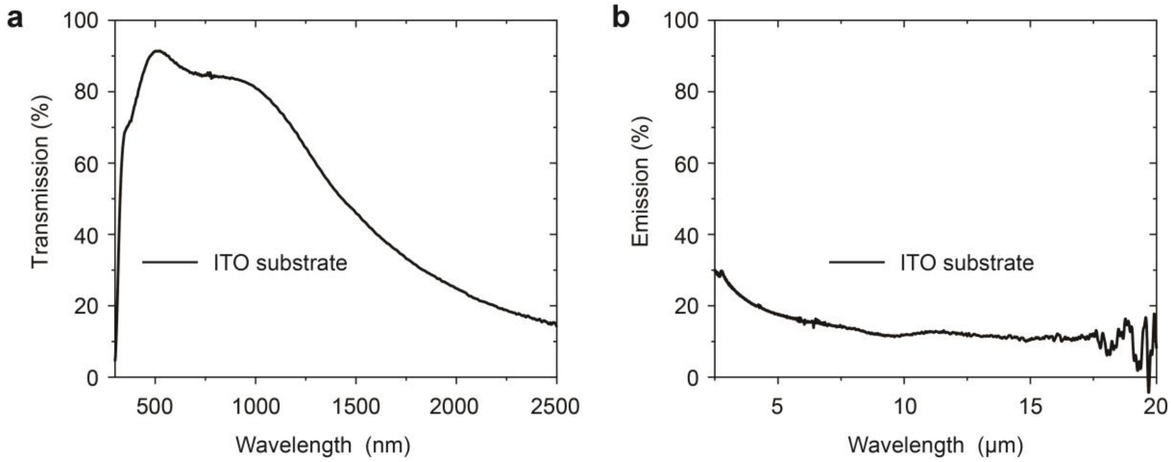

Figure. S10. The optical spectrum of the layer below 2W SMP. (a) The solar spectrum of the ITO substrate. **(b)** The mid-infrared spectrum of the ITO substrate.

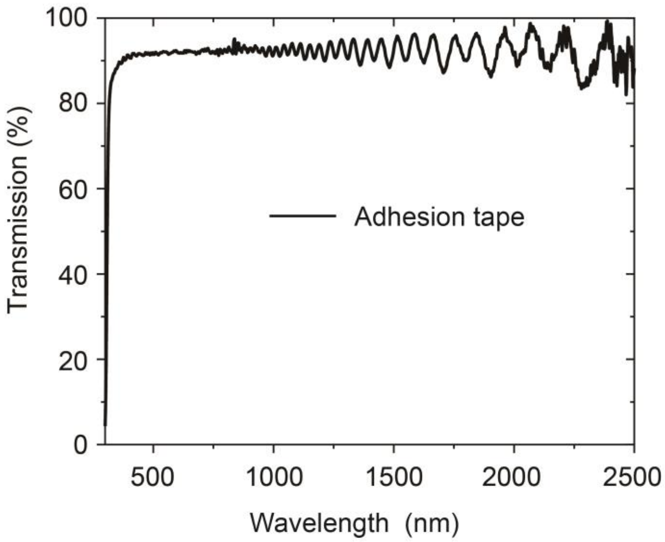

Figure. S11. The solar transmission spectrum of adhesion tape.

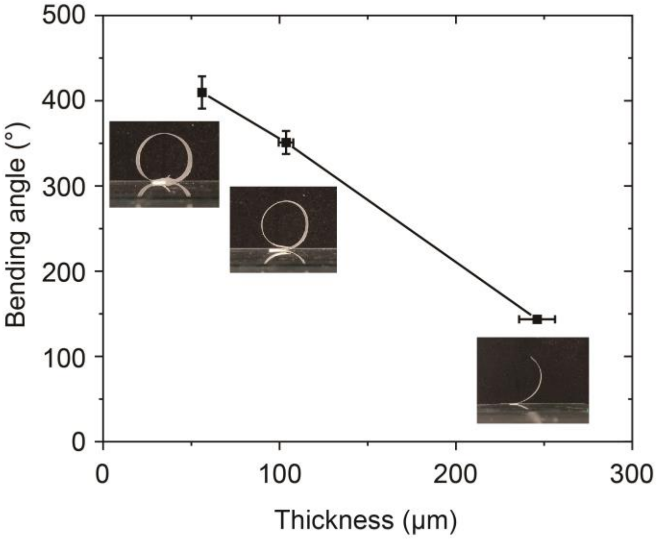

Figure. S12. The coiling ability of the device related to the thickness of 2W SMP. The 2W SMP should be limited below 100 μm to drive the upper layer to achieve at least one week of curling.

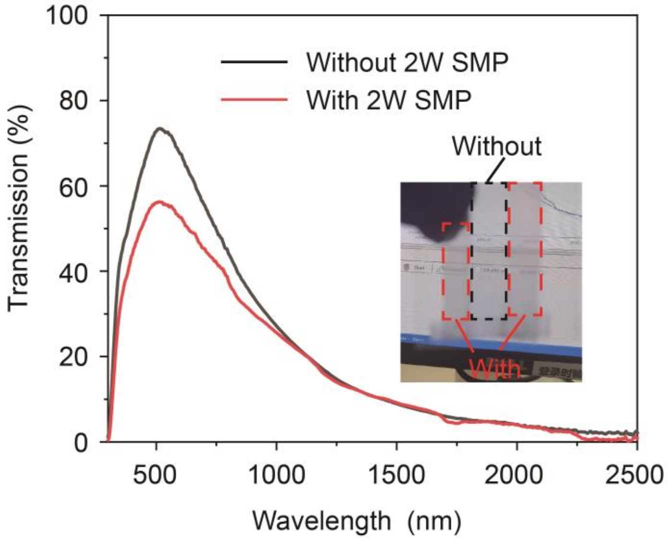

Figure. S13. The transmission spectrum of HVTW in the region with and without 2W SMP, respectively. The illustration is only used to demonstrate the 2W SMP has a haze problem that may influence the visual sense although it has a negligible effect on the solar transmissivity.

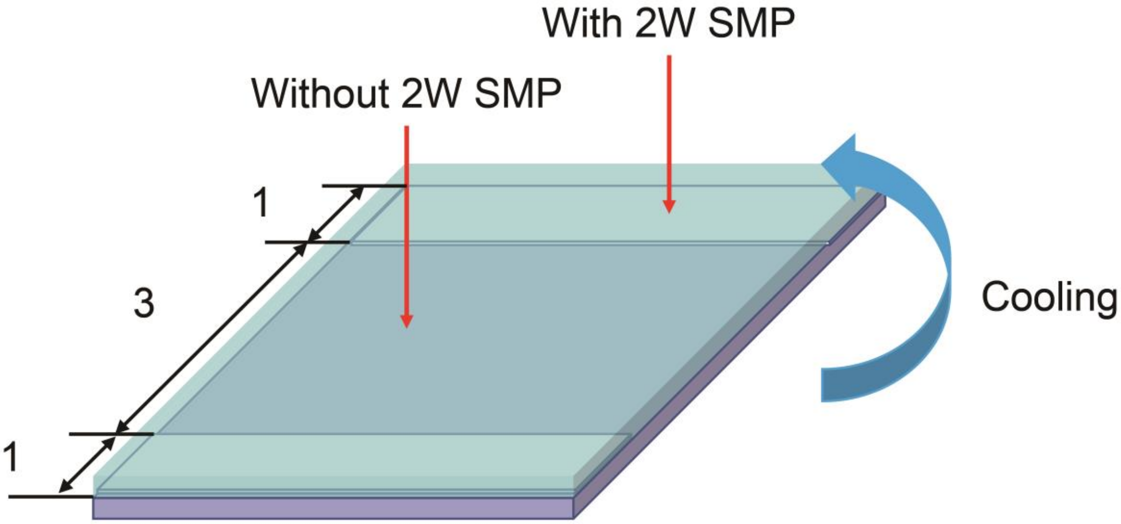

Figure. S14. The schematic of the optimized structure with spaced 2W SMP stripes for sufficient visual sense.

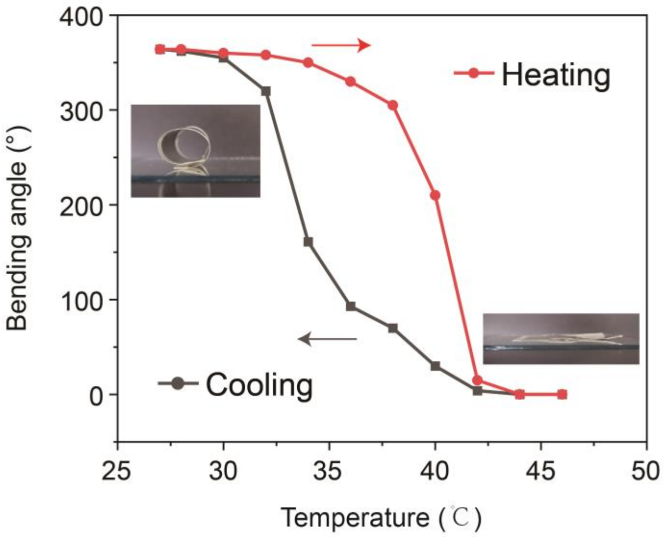

Figure. S15. Bending angle of the HVTW as a function of temperature during a heating and cooling process. The illustrations show the coiled state at 30℃ and the completely flat state at 42℃, respectively, which demonstrates that the transformation temperature of the HVTW can be adjusted.

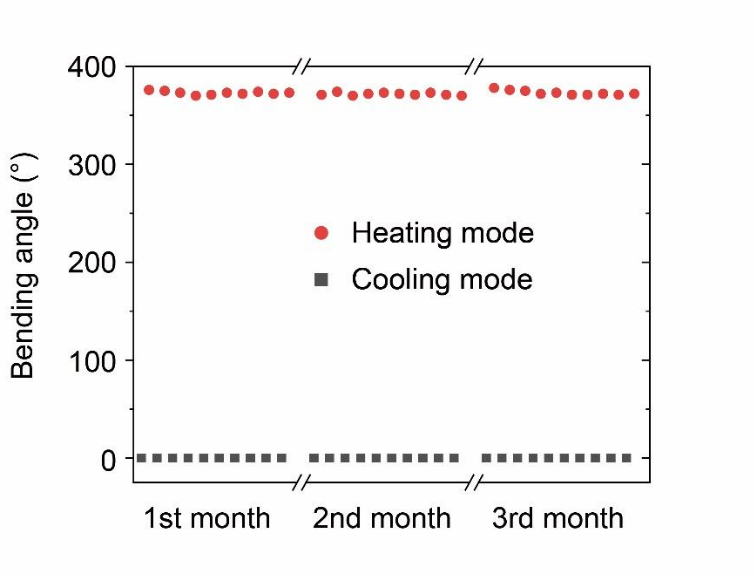

Figure. S16. Bending angle of the HVTW on the heating and cooling process for ten cycles tested every other month. It remains unchanged in 3 months, proving its excellent stability.

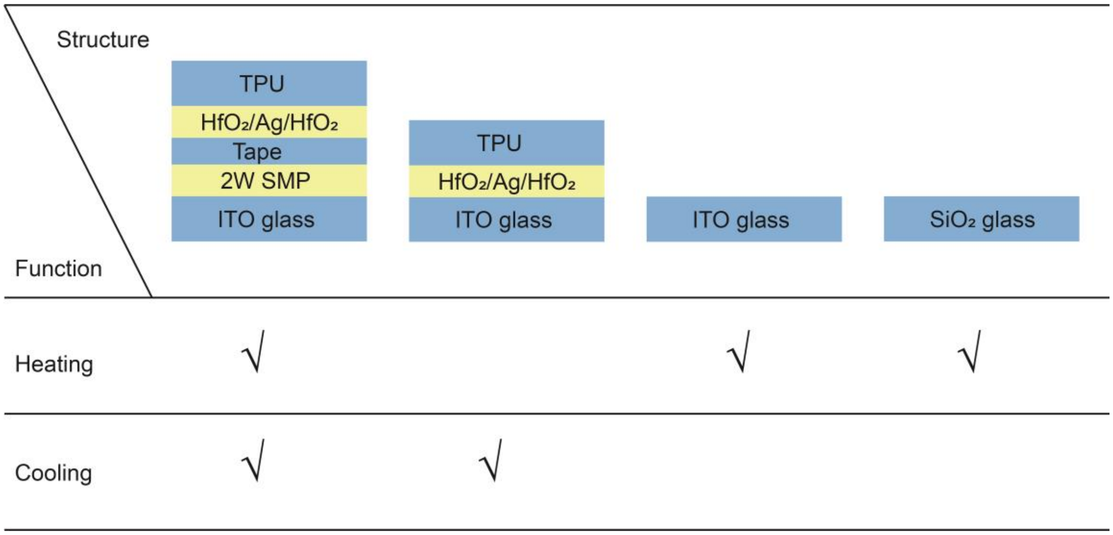

Figure. S17. The structures and functions of four control samples (the HVTW, the UVNIR sample, the ITO glass, and the SiO_2 glass, respectively) in Fig. 3.

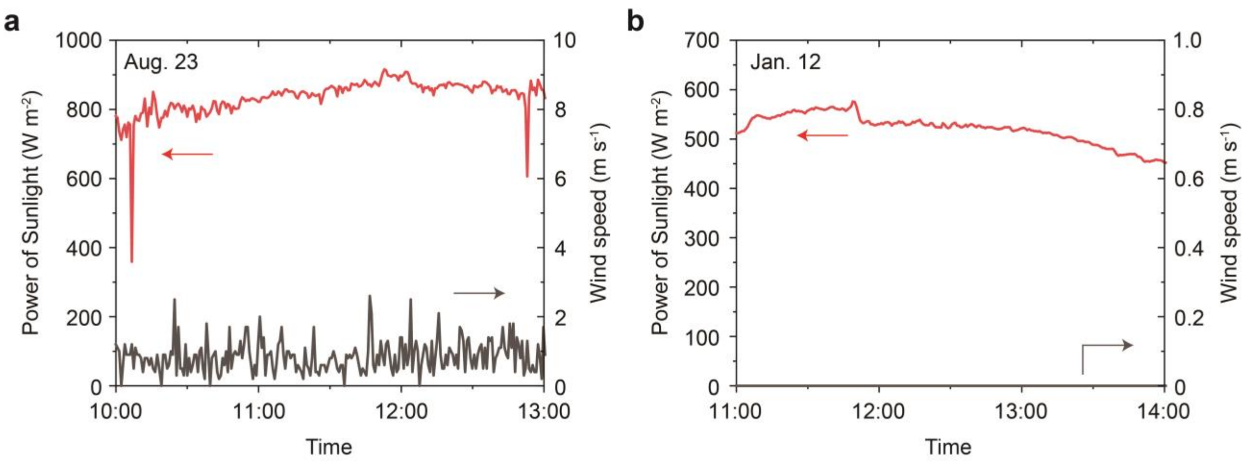

Figure. S18. (a, b) Environmental conditions of temperature tests in Fig. 3h and 3i, respectively.

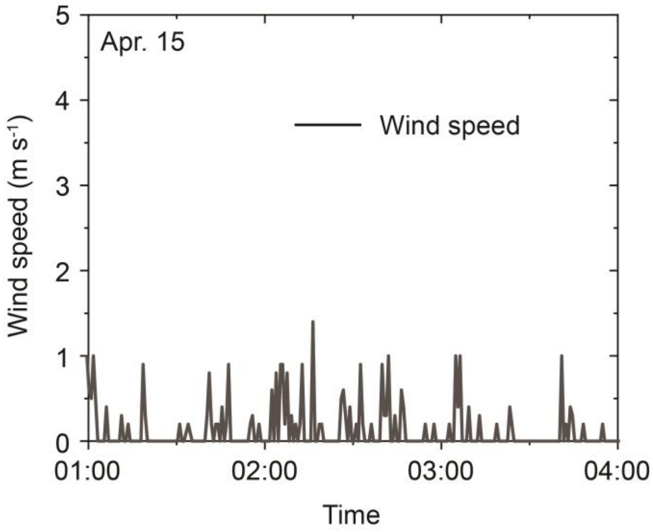

313

314 **Figure. S19. Environmental conditions of temperature tests in Fig. 3j.**

315

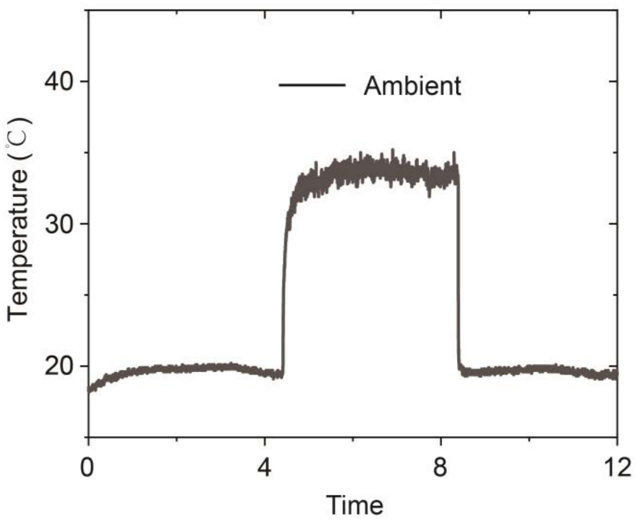

Figure. S20. The real-time ambient temperature variation in Fig. 4a.

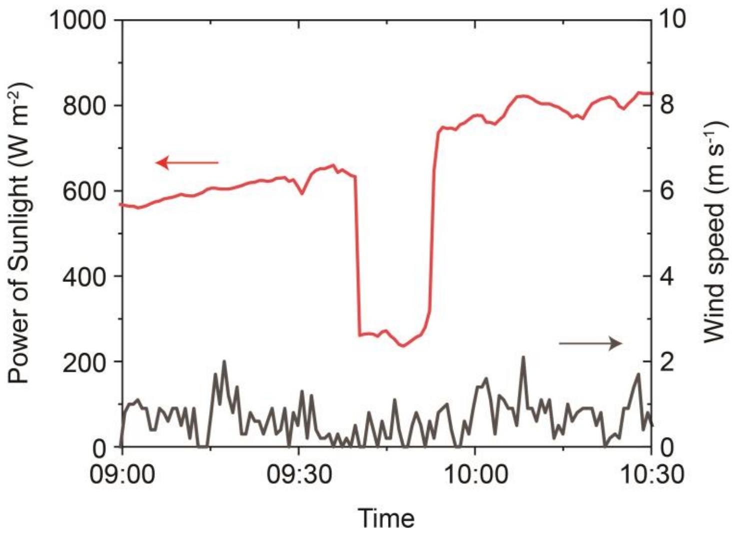

Figure. S21. Environmental conditions of temperature tests in Fig. 4b.

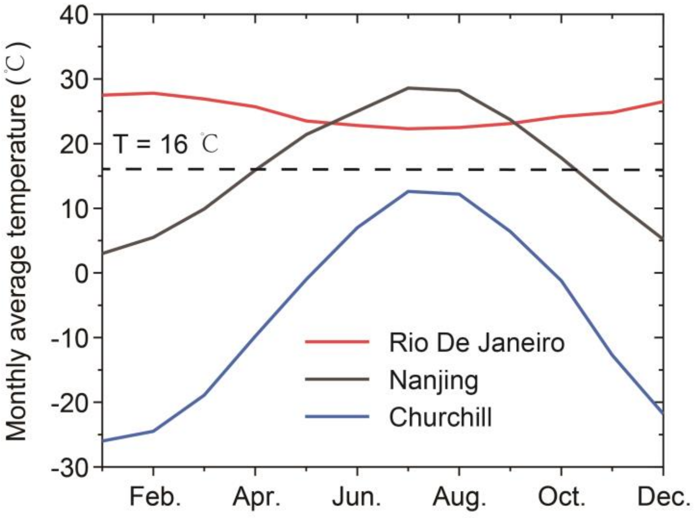

Figure. S22. The monthly average temperature of Rio De Janeiro, Nanjing, and Churchill in Fig. 4c, 4d, and 4e, respectively. The transformation temperature of the HVTW in simulation is set as 16℃.

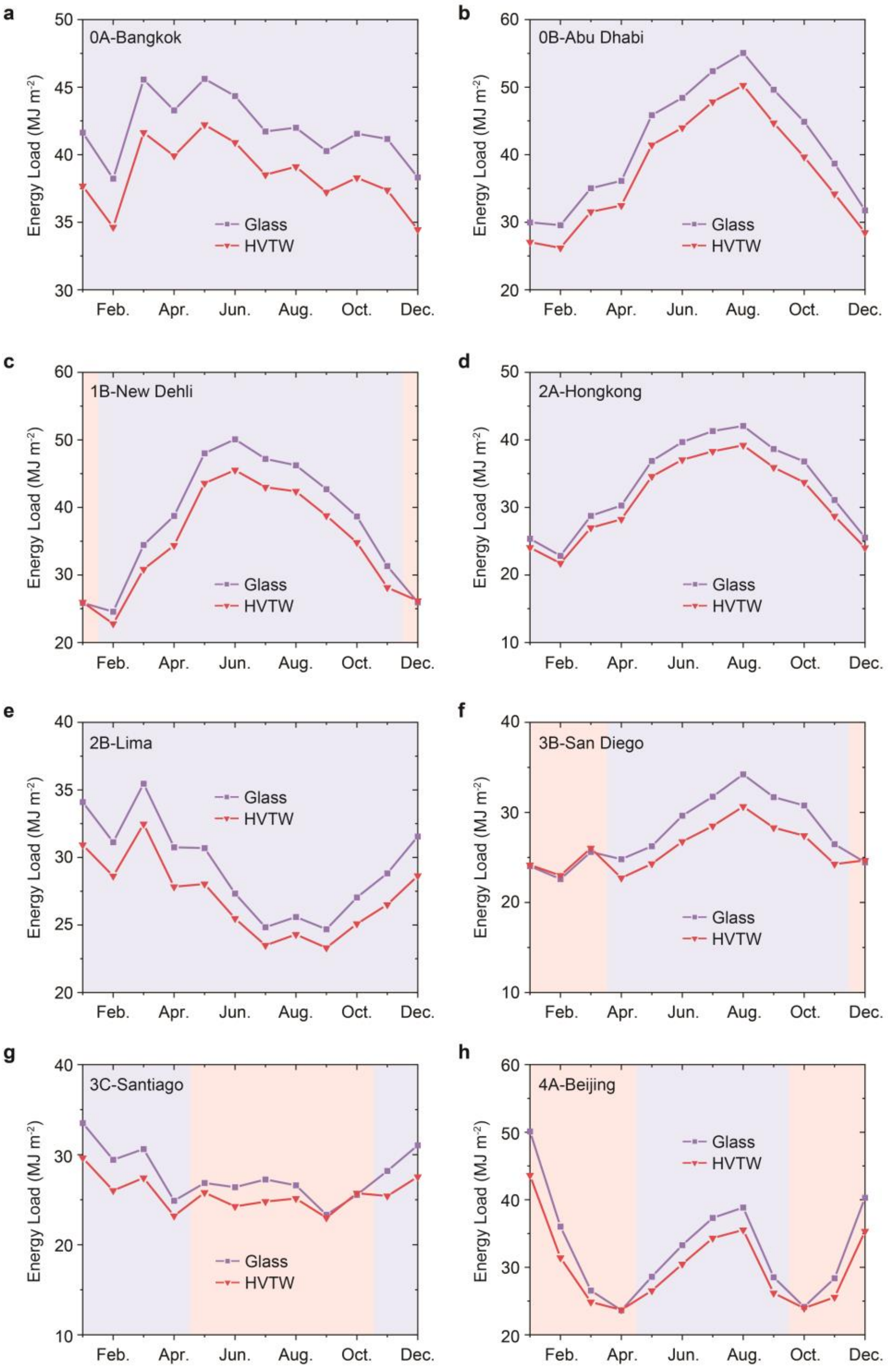

Figure. S23. Monthly energy load of the HVTW and commercial glass in typical cities in other climate zones from 0A to 4A. Our HVTW exhibits energy-saving effects for each season in all typical cities.

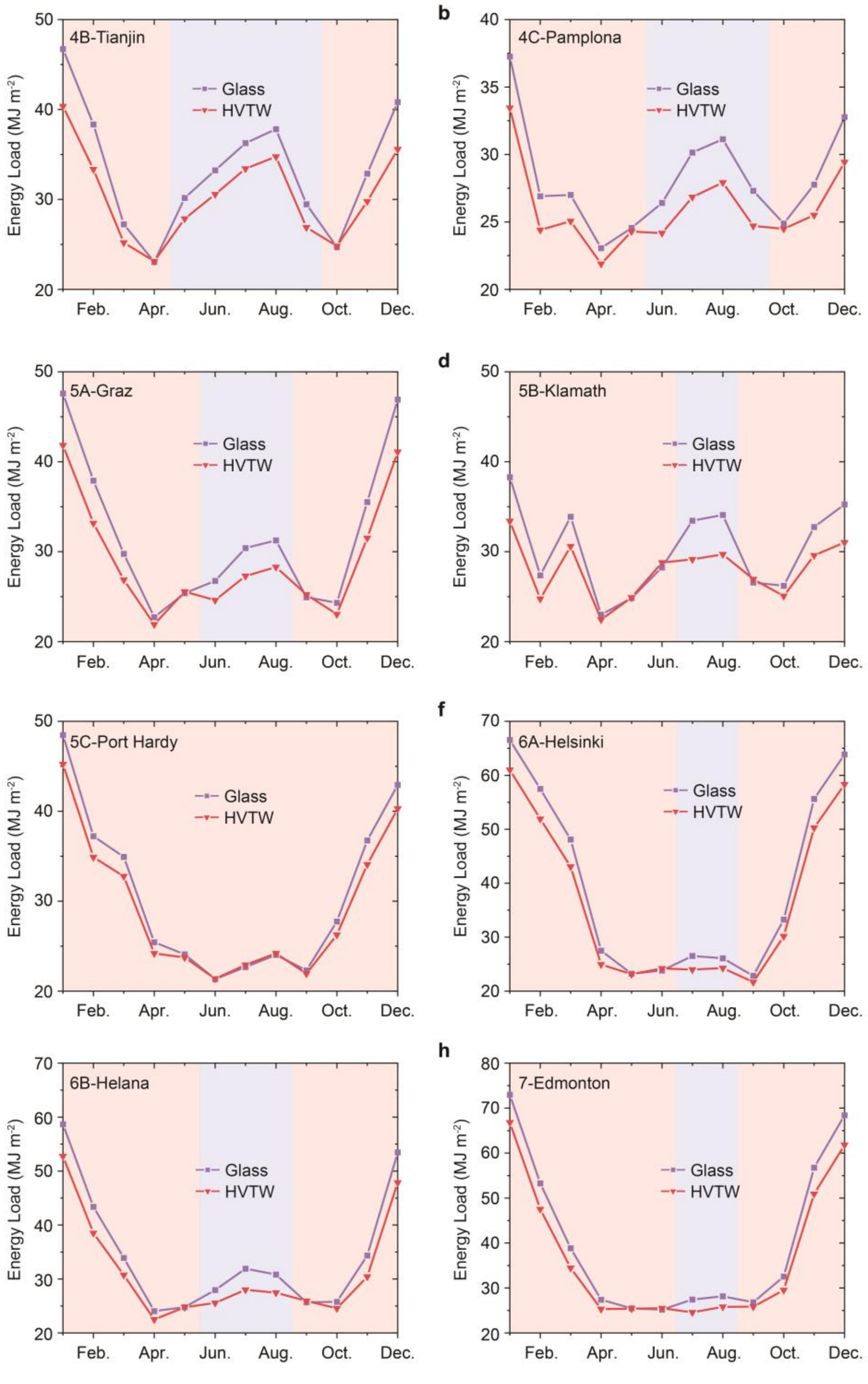

Figure. S24. Monthly energy load of the HVTW and commercial glass in typical cities in other climate zones from 4B to 7. Our HVTW exhibits energy-saving effects for each season in all typical cities.

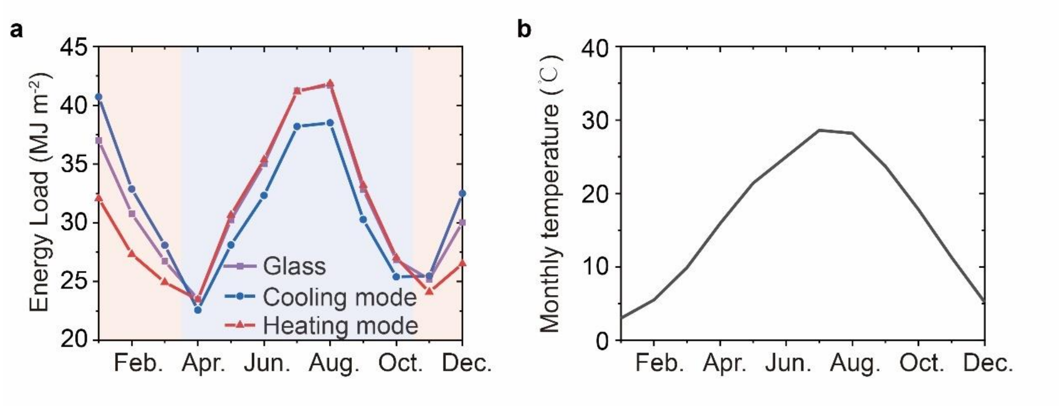

Figure. S25. The determination process of the optimal transformation temperature of the HVTW in Nanjing. (a) Monthly energy load of the cooling mode on the HVTW, the heating mode on the HVTW and commercial glass, respectively. The red shadow means that the heating mode achieves the lowest energy load while the blue shadow means that the cooling mode achieves the lowest. **(b)** The monthly average temperature of Nanjing. The optimal transformation temperature of the HVTW in Nanjing should be set within the range of monthly temperature from March to April and from September to October, which is 11.3-15.9℃.

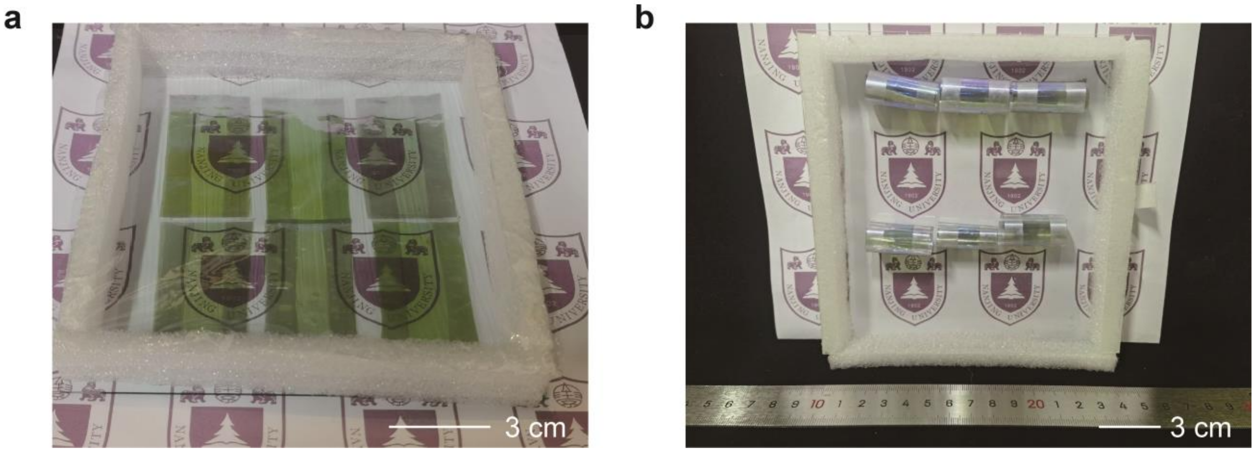

Figure. S26. The HVTW with array structure in double glazing with a spacing of 2 cm. (a) Cooling mode. **(b)** Heating mode.

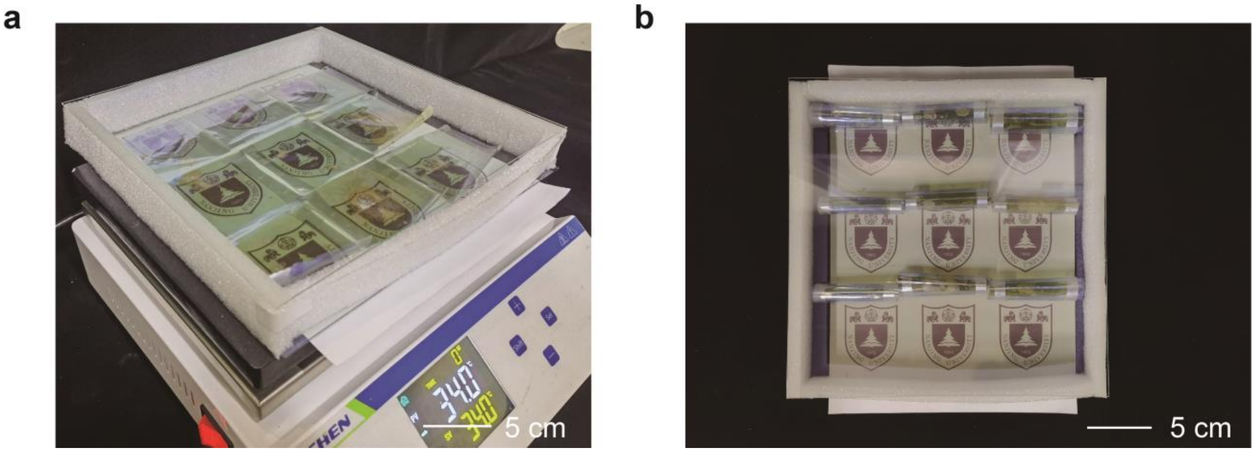

Figure. S27. The HVTW with array structure in double glazing with a spacing of 3 cm. (a) Cooling mode. **(b)** Heating mode.

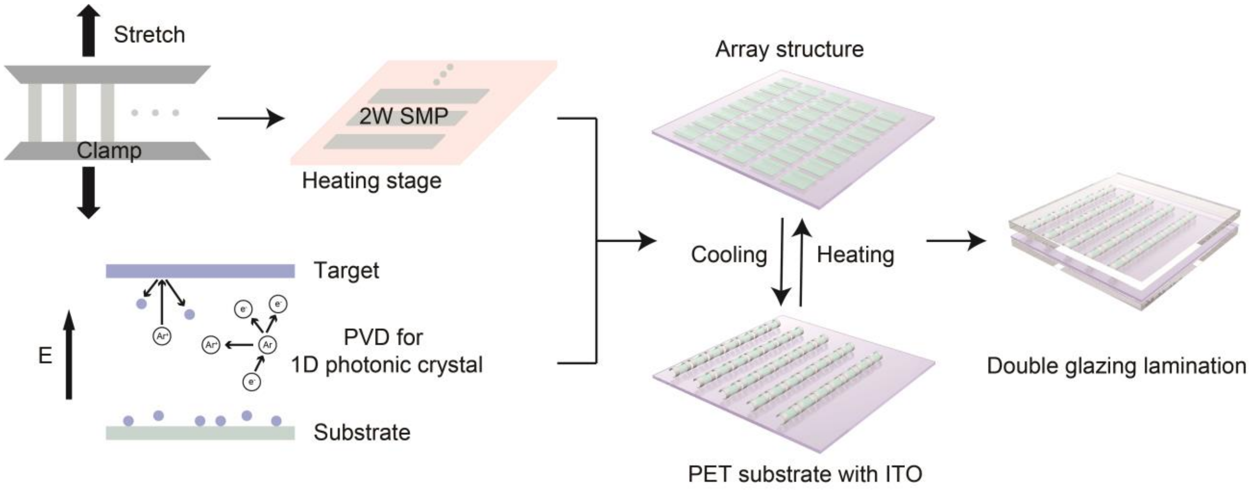

Figure. S28. Blueprint on the scalability of the HVTW. The HVTW can be further coupled with double glazing.

Tables

Table S1. Information of the medium office in energy consumption simulation.

Form	Features
Total floor area	1661.67 m^2 (49.9 m × 33.3 m)
Number of floors	3
Floor-to-floor height	4.0 m
Floor-to-ceiling height	2.7 m
Window Fraction (Window-to-wall ratio)	33%
Exterior walls	Stucco + gypsum board + wall insulation
Roof	Roof membrane + roof insulation + metal decking
Window	Based on different samples
Foundation	Slab-on-grade floor
HVAC heating type	Gas furnace inside the packaged air conditioning unit
HVAC cooling type	Packaged air conditioning unit
HVAC thermostat setpoint	23.9℃ Cooling/ 21.1℃ Heating
HVAC thermostat setback	26.7℃ Cooling/ 15.6℃ Heating

Table S2. Comparison with recent works on thermochromic smart windows.

Category	Reference	$T_{Vis\text{-}H}$	$T_{Vis\text{-}C}$	ΔT_{NIR}	$\Delta\varepsilon_{MIR}$	Trans-Tem
	**Our work**	**86.9**	**63.4**	**44.0**	**76.5**	**32℃**
VO_2	Y. Long, *et al.*, *Science*, 2021 [9]	27.8	26.1	18.6	40	90℃
	S. Yu, *et al.*, *Nat. Commun.*, 2023 [10]	66.0	67.0	25.0	0	50℃
	Y. Mei, *et al.*, *Nat. Commun.*, 2022 [11]	61.5	27.3	48.4	0	48℃
	Y. Long, *et al.*, *J. Mater. Chem. A*, 2015 [12]	82.1	43.2	29.9	0	80℃
	P. J. Klar, *et al.*, *Appl. Phys. Lett.*, 2017 [13]	61.8	61.0	10.6	0	30℃
	Z. Zhang, *et al.*, *ACS Appl. Mater. Interfaces*, 2011 [14]	59.2	61.1	16.6	0	90℃
	Y. Long, *et al.*, *J. Alloy Compd.*, 2018 [15]	47.8	45.3	26.4	0	90℃
	Y. Long, *et al.*, *Langmuir*, 2014 [16]	43.6	45.3	19.4	0	90℃
	M. Kanehira, *et al.*, *Energ. Environ. Sci.*, 2012 [17]	53.0	49.8	22.1	0	90 ℃
	P. Jin, *et al.*, *New. J. Chem.*, 2017 [18]	73.36	68.71	16.9	0	90℃
Hydrogel	B. Huang, *et al.*, *Sci. Adv.*, 2022 [19]	78.3	7.0	42.6	57.1	32℃
	J. Fu, *et al.*, *Adv. Mater.*, 2023 [20]	91.3	0	85.7	14	45℃
	X. Fang, *et al.*, *Joule*, 2019 [21]	87.2	1.3	75.6	0	34℃
	Y. Long, *et al.*, *Nano*	71.6	7.8	33.7	85	40℃

	Energy, 2021 [22]					
	Y. Long, *et al.*, *RCS. Adv.*, 2016 [23]	88.4	87.5	0.3	0	45℃
	Y. Long, *et al.*, *RCS. Adv.*, 2016 [23]	82.5	15.6	30.9	0	45℃
	Y. Long, *et al.*, *J. Mater. Chem. A*, 2014 [24]	87.9	59.9	9.5	0	40℃
	L. Wang, *et al.*, *Nat. Commun.*, 2018 [25]	64.0	0	70.7	0	40℃
	Y. Long, *et al.*, *Adv. Funct. Mater.*, 2018 [26]	81.0	1.4	64.2	0	60℃
Ionic liquid	P. Jin, *et al.*, *ACS Appl. Mater. Interfaces*, 2016 [27]	66.9	51.9	35.0	0	80℃
	X. M. Hu, *et al.*, *Chem. Mater.*, 2017 [28]	87.8	7.7	55.4	0	60℃
Perovskite	K. M. Yu, *et al.*, *Appl. Energ.*, 2019 [29]	89.1	36.0	0	0	80℃
Liquid crystal	H. Yang, *et al.*, *Mater. Horiz.*, 2017 [30]	77.8	2.9	45.2	0	30℃
Thermo-mechanochromic	Y. Long, *et al.*, *Joule*, 2019 [31]	35.2	17.6	64.0	0	95℃
Photo-thermochromic	S. Cheng, *et al.*, *Sol. RRL*, 2018 [32]	88.9	3.5	36.0	0	40℃
	S. Cheng, *et al.*, *Sol. RRL*, 2018 [32]	88.9	38.4	6.9	0	40℃
	L. Wang, *et al.*, *Sol. RRL*, 2018 [33]	77.2	5.0	36.0	0	40℃
	P. Wang, *et al.*, *ACS Appl. Mater. Interfaces*, 2018 [34]	78.0	44.9	6.5	0	36℃

* T_{Vis-H}: Visible transmissivity in heating mode; T_{Vis-C}: Visible transmissivity in cooling mode.
Trans-Tem: Transition temperature.

Table S3. Optical properties for different samples in energy consumption simulation.

Optical properties	Glass	HVTW-Cooling mode	HVTW-Heating mode
Solar transmittance	0.8	0.452	0.776
Front-side solar reflectance	0.1	0.473	0.149
Back-side solar reflectance	0.1	0.075	0.075
Visible transmittance	0.8	0.634	0.869
Front side visible reflectance	0.1	0.276	0.081
Back side visible reflectance	0.1	0.081	0.081
Infrared transmittance	0	0	0
Front-side infrared emissivity	0.8	0.887	0.123
Back-side infrared emissivity	0.8	0.8	0.8

Table S4. Average annual energy saving with the HVTW in 19 climate zones globally against a commercial glass as the baseline.

Region	Thermal zone	Thermal climate zone name	Annual energy saving (MJ m^{-2})
Bangkok metropolis, Thailand	0A	Extremely hot humid	41.8
Abu Dhabi Intl, United Arab Emirates	0B	Extremely Hot Dry	49.6
Rio De Janeiro galeao, Brazil	1A	Very Hot Humid	38.3
New Delhi Indira Gandhi Intl, India	1B	Very Hot Dry	37.4
Hong Kong Cheung Chau, China	2A	Hot Humid	26.8
Lima, Peru	2B	Hot Dry	27.3
Nanjing, China	3A	Warm Humid	29.9
San Diego, Califonia, United States	3B	Warm Dry	21.6
Santiago Pudahuel, Chile	3C	Warm Marine	25.7
Beijing, China	4A	Mixed Humid	34.3
Tianjin, China	4B	Mixed Dry	35.0
Pamplona, Spain	4C	Mixed Marine	27.0
Graz, Austria	5A	Cool Humid	33.1
Klamath, Oregon, United States	5B	Cool Dry	27.5
Port Hardy, British Columbia, Canada	5C	Cool Marine	16.0
Helsinki Harmaja, Finland	6A	Cold Humid	37.9
Helana, Montana, United States	6B	Cold Dry	35.7
Edmonton Intl, Alberta, Canada	7	Very Cold	39.7
Churchill, Manitoba, Canada	8	Subarctic/Arctic	40.6

Movies

Movie S1. The dynamic process of the HVTW from the cooling mode to the heating mode. The video is played at 5x speed.

Movie S2. Dynamic process of the HVTW from heating mode to cooling mode. The video is played at 5x speed.

References

1. ANSI/ASHRAE/IES Standard 90.1. *Prototype Building Models*. https://www.energycodes.gov/prototype-building-models#ASHRAE (9 November 2024, date last accessed).
2. Winiarski DW, Halverson MA, Jiang W. Analysis of building envelope construction in 2003 CBECS. Pacific Northwest National Lab, Richland, United States, 2007.
3. Goel S, Michael R, Rahule A *et al.* Enhancements to ASHRAE standard 90.1 prototype building models. Pacific Northwest National Lab, Richland, United States, 2014.
4. ANSI/ASHRAE Standard 169-2013; *Climatic data for building design standards*. https://store.accuristech.com/ashrae/standards/ashrae-169-2013?product_id=1869436 (9 November 2024, date last accessed).
5. ANSI/ASHRAE Standard 169-2020; *Climatic data for building design standards*. https://store.accuristech.com/ashrae/standards/ashrae-169-2020?product_id=2195141 (9 November 2024, date last accessed).
6. Somasundaram S, Chong A, Wei Z *et al.* Energy saving potential of low-e coating based retrofit double glazing for tropical climate. *Energ Buildings* 2020; **206**: 109570.
7. He Q, Ng ST, Hossain MU *et al.* Energy-efficient window retrofit for high-rise residential buildings in different climatic zones of China. *Sustainability* 2019; **11**: 6473.
8. Yaşar Y and Kalfa SM. The effects of window alternatives on energy efficiency and building economy in high-rise residential buildings in moderate to humid climates. *Energy Convers Manage* 2012; **64**: 170-81.
9. Wang S, Jiang T, Meng Y *et al.* Scalable thermochromic smart windows with passive radiative cooling regulation. *Science* 2021; **374**: 1501-4.
10. Sheng SZ, Wang JL, Zhao B *et al.* Nanowire-based smart windows combining electro-and thermochromics for dynamic regulation of solar radiation. *Nat Commun* 2023; **14**: 3231.
11. Li X, Cao C, Liu C *et al.* Self-rolling of vanadium dioxide nanomembranes for enhanced multi-level solar modulation. *Nat Commun* 2022; **13**: 7819.
12. Zhou Y, Cai Y, Hu X *et al.* VO_2/hydrogel hybrid nanothermochromic material with ultra-high solar modulation and luminous transmission. *J Mater Chem A* 2015; **3**: 1121-6.
13. Dietrich MK, Kuhl F, Polity A *et al.* Optimizing thermochromic VO_2 by co-doping with W and Sr for smart window applications. *Appl Phys Lett* 2017; **110**: 141907.

14. Kang L, Gao Y, Luo H *et al.* Nanoporous thermochromic VO_2 films with low optical constants, enhanced luminous transmittance and thermochromic properties. *ACS Appl Mater Interfaces* 2011; **3**: 135-8.
15. Liu C, Wang S, Zhou Y *et al.* Index-tunable anti-reflection coatings: Maximizing solar modulation ability for vanadium dioxide-based smart thermochromic glazing. *J Alloys Compd* 2018; **731**: 1197-207.
16. Qian X, Wang N, Li Y *et al.* Bioinspired multifunctional vanadium dioxide: improved thermochromism and hydrophobicity. *Langmuir* 2014; **30**: 10766-71.
17. Gao Y, Wang S, Kang L *et al.* VO_2-Sb: SnO_2 composite thermochromic smart glass foil. *Energ Environ Sci* 2012; **5**: 8234-7.
18. Zhu J, Huang A, Ma H *et al.* Hybrid films of VO_2 nanoparticles and a nickel(Ⅱ)-based ligand exchange thermochromic system: excellent optical performance with a temperature responsive colour change. *New J Chem* 2017; **41**: 830-5.
19. Lin C, Hur J, Chao CY *et al.* All-weather thermochromic windows for synchronous solar and thermal radiation regulation. *Sci Adv* 2022; **8**: eabn7359.
20. Chen G, Wang K, Yang J *et al.* Printable thermochromic hydrogel-based smart window for all-weather building temperature regulation in diverse climates. *Adv Mater* 2023; **35**: 2211716.
21. Li XH, Liu C, Feng SP *et al.* Broadband light management with thermochromic hydrogel microparticles for smart windows. *Joule* 2019; **3**: 290-302.
22. Wang S, Zhou Y, Jiang T *et al.* Thermochromic smart windows with highly regulated radiative cooling and solar transmission. *Nano Energy* 2021; **89**: 106440.
23. Yang YS, Zhou Y, Chiang FBY *et al.* Temperature-responsive hydroxypropylcellulose based thermochromic material and its smart window application. *RSC Adv* 2016; **6**: 61449-53.
24. Zhou Y, Cai Y, Hu X *et al.* Temperature-responsive hydrogel with ultra-large solar modulation and high luminous transmission for “smart window” applications. *J Mater Chem A* 2014; **2**: 13550-5.
25. Wang S, Xu Z, Wang T *et al.* Warm/cool-tone switchable thermochromic material for smart windows by orthogonally integrating properties of pillar[6]arene and ferrocene. *Nat Commun* 2018; **9**: 1737.

26. Zhou Y, Layani M, Wang S *et al.* Fully printed flexible smart hybrid hydrogels. *Adv Funct Mater* 2018; **28**: 1705365.

27. Zhu J, Huang A, Ma H *et al.* Composite film of vanadium dioxide nanoparticles and ionic liquid-nickel-chlorine complexes with excellent visible thermochromic performance. *ACS Appl Mater Interfaces* 2016; **8**: 29742-8.

28. Lee HY, Cai Y, Velioglu S *et al.* Thermochromic ionogel: a new class of stimuli responsive materials with super cyclic stability for solar modulation. *Chem Mater* 2017; **29**: 6947-55.

29. Zhang Y, Tso C, Iñigo JS *et al.* Perovskite thermochromic smart window: Advanced optical properties and low transition temperature. *Appl Energ* 2019; **254**: 113690.

30. Liang X, Guo S, Chen M *et al.* A temperature and electric field-responsive flexible smart film with full broadband optical modulation. *Mater Horiz* 2017; **4**: 878-84.

31. Ke Y, Yin Y, Zhang Q *et al.* Adaptive thermochromic windows from active plasmonic elastomers. *Joule* 2019; **3**: 858-71.

32. Cao D, Xu C, Lu W *et al.* Sunlight-driven photo-thermochromic smart windows. *Sol RRL* 2018; **2**: 1700219.

33. Xu Z, Wang S, Hu XY *et al.* Sunlight-induced photo-thermochromic supramolecular nanocomposite hydrogel film for energy-saving smart window. *Sol RRL* 2018; **2**: 1800204.

34. Wu M, Shi Y, Li R *et al.* Spectrally selective smart window with high near-infrared light shielding and controllable visible light transmittance. *ACS Appl Mater Interfaces* 2018; **10**: 39819-27.
